# SportRGDET: Scene prior and relation graph enhanced detection transformer for sports scenes

**DOI:** 10.1371/journal.pone.0348924

**Published:** 2026-10-16

**Authors:** Qingya Ouyang, Chaoyi Su, Qingyuan Li

**Affiliations:** 1 Department of Physical Education, Xiamen University, Xiamen, Fujian, China; 2 Department of Physical Education, Sun Yat-sen University, Guangzhou, Guangdong, China; 3 Sports Culture Research Center, Tianjin University of Sport, Tianjin, China; National University of Singapore, SINGAPORE

## Abstract

Object detection in sports videos holds significant application value in tactical analysis, athlete tracking, and intelligent broadcasting. However, existing methods face two key challenges when processing sports scenes: on one hand, target distribution in sports footage is strictly constrained by field boundaries, yet generic detectors uniformly allocate detection resources across the entire image, causing frequent false detections in off-field regions due to the visual similarity between spectators and athletes; on the other hand, objects such as athletes, balls, and referees in sports scenes form stable spatial co-occurrence patterns governed by game rules, yet existing methods predict each object independently, failing to leverage cross-category relational cues to assist the detection of difficult targets. To address these issues, this paper proposes SportRGDET (Sport Relation-Graph DEtection Transformer), a framework that achieves accurate detection through two core modules: the Scene Prior Guided Module (SPGM) jointly estimates the spatial probability distribution and boundary gradient map of the field region from multi-scale encoder features, fuses them to generate a hierarchical field confidence mask, and performs spatial modulation on the deformable self-attention weights of the encoder, which *reallocates* attention resources toward the effective field area and progressively suppresses off-field interference across layers, thereby reducing false detections while incurring only moderate additional computation; the Relation Reasoning Enhancement Module (RREM) treats each candidate object as a node during the detection decoding stage, adaptively learns inter-node association strengths through a relation encoder based on category embeddings and relative spatial positions of node pairs, constructs a dynamic relation graph, and bidirectionally propagates contextual information across nodes via graph attention mechanisms, enabling difficult targets to refine their predictions by leveraging positional and categorical cues from surrounding identified objects, enhancing detection robustness under occlusion and blur conditions. Experimental results demonstrate that SportRGDET achieves significant performance improvements on multiple sports detection benchmark datasets, validating the effectiveness of scene prior and relation reasoning mechanisms in sports scene object detection.

## Introduction

Object detection in sports videos is an important research direction in computer vision with broad application value in intelligent sports analysis. With the accelerating digital transformation of the sports industry, the growing demand for automated athlete tracking, tactical analysis, highlight extraction, and intelligent broadcasting has placed urgent requirements on the accurate detection of multiple object categories including athletes, balls, and referees in video. Unlike general natural scenes, sports scenes possess distinctive domain characteristics: the playing field provides explicit spatial boundary constraints, stable spatial associations determined by game rules exist between different object categories, and target distributions exhibit highly structured patterns. As illustrated in Fig 1, these domain characteristics contain rich prior knowledge that can theoretically provide powerful guidance for detection algorithms. However, most existing methods directly adopt generic object detection frameworks without effectively exploiting the structured properties of sports scenes, resulting in limited performance in practical applications.

**Fig 1:**
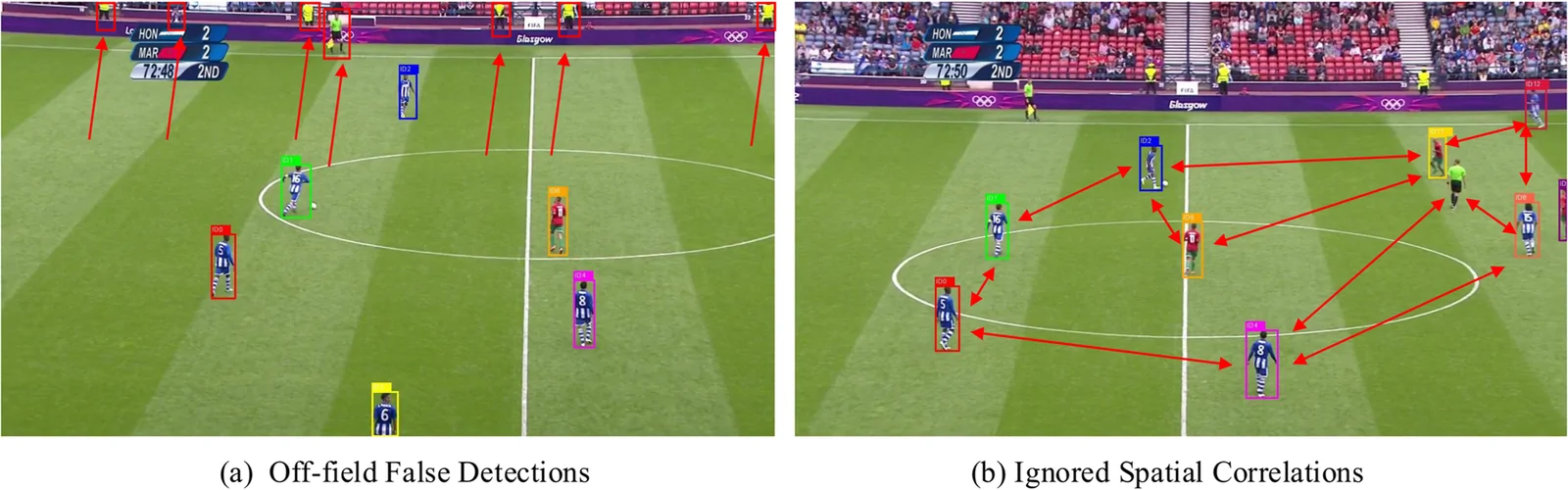
Two key challenges in sports object detection. (a) Without field-aware spatial constraints, detectors allocate attention uniformly across the entire image, causing off-field personnel (e.g., spectators, staff) to be falsely detected as players due to their similar human appearance. Red arrows indicate false positive detections in off-field regions. (b) Objects in sports scenes exhibit strong spatial correlations governed by game rules, such as the ball remaining within players’ reachable range and referees maintaining proximity to contested areas (indicated by red arrows). However, existing detectors predict each object independently, failing to leverage these cross-object spatial relations to improve detection of occluded or blurred targets.

Current sports object detection methods suffer from two core technical bottlenecks that severely constrain their practical performance. First, regarding attention allocation, existing generic detectors fail to exploit the inherent spatial structural constraints of sports scenes. Target distribution in sports footage is highly non-uniform: athletes are strictly confined within field boundaries, while off-field regions (spectator stands, billboards, substitute benches) occupy the majority of the frame area but contain no detection targets. Mainstream methods such as DETR distribute learnable queries uniformly across the entire image, and the YOLO series places anchors at all grid positions, allocating identical attention capacity to both on-field and off-field regions. This scene-agnostic detection strategy leads to frequent false detections in off-field regions, because spectators in the stands share highly similar appearance with on-field athletes (both being human figures wearing similar sportswear), and detectors lacking spatial constraints thus severely degrade precision. Second, regarding prediction mechanisms, existing methods detect each object independently, ignoring the strong spatial associations among different object categories in sports scenes. In sports matches, objects such as athletes, balls, and referees do not exist in isolation but form stable spatial co-occurrence patterns governed by game rules and physical dynamics. For example, the football always remains within players’ reachable range, referees maintain fixed distances from the nearest contested area, and goalkeepers are confined to the vicinity of the goal area. However, the prediction heads of detectors such as Faster R-CNN and DETR perform independent regression for each object, completely discarding these cross-category relational cues. Such isolated prediction is acceptable when targets are clearly visible, but when objects are difficult to identify solely from their own appearance due to motion blur or occlusion, the lack of relational reasoning capability directly leads to missed detections. For instance, a blurred football could potentially have its position inferred from the orientations of surrounding players, but the isolated prediction mechanism cannot achieve such cross-object reasoning.

To address these challenges, we propose SportRGDET (Sport Relation-Graph DEtection Transformer), a sports scene object detection framework that integrates scene spatial priors with cross-object relation reasoning. The core idea of this framework is to explicitly model and inject two types of domain priors from sports scenes, namely field structure and inter-object spatial associations, into the detection pipeline, guiding the detector’s spatial attention and reasoning behavior during the feature encoding and object decoding stages, respectively. The framework contains two core modules: the Scene Prior Guided Module (SPGM) and the Relation Reasoning Enhancement Module (RREM). Specifically, SPGM attaches a field-aware branch to the backbone’s multi-scale features, predicting pixel-level field probability maps through semantic segmentation and extracting field boundary response maps using a learnable differential operator. The two are fused via adaptive integration to generate a field confidence mask. This mask performs multiplicative modulation on the encoder’s deformable self-attention weights (following the Deformable DETR encoder design, in which self-attention is used inside the encoder while cross-attention appears in the decoder), *reallocating* feature responses toward within-field regions and progressively down-weighting off-field regions across layers. Rather than pruning tokens or reducing FLOPs, SPGM regularizes where attention is spent, achieving an accuracy and overhead trade-off that is quantified in the experiments. The field-aware branch is trained through auxiliary segmentation supervision and adds only minimal computational overhead during inference. RREM organizes all candidate objects output by the decoder into a dynamic graph structure, with each candidate object serving as a node whose features comprise the object embedding, spatial position encoding, and category probability. Edge weights between nodes are adaptively learned by a relation encoder that takes category embeddings and relative spatial positions of node pairs as input and predicts inter-node association strengths, enabling the network to learn spatial association patterns between different object categories in sports scenes in an end-to-end manner. On this graph, a multi-layer graph attention network propagates information between nodes, allowing each candidate object’s representation to incorporate context from associated objects, thereby enabling occluded or blurred targets to achieve more accurate localization and classification by leveraging cues from surrounding identified objects. Consistent with recent evidence that heterogeneous representation alignment should be calibrated by task-relevant shared information rather than maximized indiscriminately [1], our gated relation encoder deliberately performs *selective* rather than unconditional fusion between geometric and categorical branches. Experiments on multiple sports detection benchmarks demonstrate that SportRGDET achieves significant improvements in mAP@0.5 over the baseline Deformable DETR, with SPGM and RREM each contributing distinct and complementary performance gains. Additional leave-one-sport-out and cross-dataset transfer experiments further characterize the method’s out-of-distribution behavior.

The main contributions of this paper are as follows:

1. We propose the Scene Prior Guided Module (SPGM), which generates confidence masks from field probability maps and boundary response maps to spatially modulate the encoder’s deformable self-attention, reallocating attention toward the field area and thus reducing off-field false detections at the feature level rather than through post-processing.
2. We design the Relation Reasoning Enhancement Module (RREM), which learns inter-object spatial association patterns end-to-end through a relation encoder and constructs dynamic graph structures, leveraging graph attention to achieve cross-object context propagation and breaking through the limitations of isolated per-object prediction.
3. Experiments on three sports detection benchmark datasets, controlled comparisons against auxiliary-supervision baselines with matched annotation budgets, leave-one-sport-out generalization, and edge-case robustness analysis together validate the effectiveness of SportRGDET and delineate its scope of applicability.

## Related work

### Object detection in sports scenes

Object detection has evolved from two-stage detectors (Faster R-CNN [2]) to single-stage detectors (YOLO [3]) and further to Transformer-based end-to-end detectors. DETR [4] first introduced Transformers into object detection, eliminating the dependence on hand-crafted post-processing such as NMS through learnable queries and bipartite matching, but suffers from slow convergence and insufficient performance on small objects. Subsequently, Deformable DETR [5] introduced deformable attention to reduce the training period to one-tenth, DAB-DETR [6] parameterized queries as dynamic anchor boxes, DN-DETR [7] accelerated matching convergence through denoising training, and DINO [8] integrated the above improvements to achieve a leading 63.3 AP on COCO.

In sports scenes, the detection task faces special challenges including large target scale variations, fast motion, and frequent occlusion. Cioppa et al. [9] released the SoccerNet-Tracking dataset covering football videos from multiple European leagues, providing a large-scale benchmark for player detection and tracking. Cui et al. [10] constructed the SportsMOT dataset, extending multi-object tracking to multiple sports including football and basketball. Regarding ball detection, Moreira et al. [11] surveyed ball detection literature from 2021 to 2024, identifying occlusion and small object recall as common bottlenecks across sports. In terms of field awareness, Homayounfar et al. [12] proposed a field localization method based on deep structured models, and Nie et al. [13] further combined semantic segmentation with homography estimation to achieve accurate field registration. However, most existing sports detection methods directly adopt generic detectors without explicitly incorporating scene spatial structural constraints and inter-object relational associations into the detection pipeline.

### Scene prior and spatially-guided detection

Attention mechanisms serve as an important means to guide detectors toward effective regions. SE-Net [14] learns inter-channel dependencies through global average pooling to recalibrate channel-level feature responses, but ignores spatial dimension information. CBAM [15] builds upon this by introducing a spatial attention sub-module, sequentially inferring attention maps along the channel and spatial dimensions for feature modulation. Regarding scene prior guidance, Cao et al. [16] proposed a mask-guided feature modulation method that uses semantic segmentation masks to guide feature fusion from both global and local perspectives, enabling detectors to focus on target regions. In sports scenes, the field region provides a natural spatial prior. Existing field segmentation methods [12,13] can localize the field region but typically operate as independent preprocessing steps without deep coupling with the detector’s feature encoding. From a representation-alignment perspective, Fang et al. [1] recently argued that alignment between heterogeneous modalities (or streams) should be adaptively calibrated by the amount of shared and source-specific task information rather than being forced to a maximum; this motivates the gated integration used inside our relation encoder as well as the adaptive dual-stream fusion in SPGM. Unlike the above works, our SPGM generates hierarchical masks by jointly estimating field probability maps and boundary gradient maps, and performs spatial modulation directly on the encoder’s deformable self-attention layers to achieve end-to-end fusion of scene priors with detection features.

### Relation reasoning and graph-based detection

Relation reasoning aims to leverage contextual associations among objects to improve detection performance. Hu et al. [17] proposed Relation Networks, learning inter-object association weights through the interaction of appearance features and geometric positions, but the fully-connected relation modeling introduces redundant noisy connections when the number of objects is large. Xu et al. [18] proposed SGRN, which adaptively learns sparse adjacency matrices through a graph learning module and introduces spatial Gaussian kernels to encode pairwise spatial information, achieving significant improvements in large-scale detection. RGRN [19] further develops relation-aware graph reasoning, enhancing detection capability under occlusion and semantic confusion. In the visual relation modeling domain, VTransE [20] models inter-object semantic relations through vector translation, and Neural Motifs [21] encodes global context in scene graphs through bidirectional LSTMs. GCN [22] and GAT [23] provide message passing paradigms that lay the foundation for inter-object information propagation. However, existing methods are mostly designed for generic scenes without considering spatial co-occurrence patterns determined by rule constraints in specific domains, and relation reasoning typically serves as a post-processing step rather than being deeply integrated with the decoder. Our RREM targets the cross-category spatial associations unique to sports scenes, constructing dynamic relation graphs during the decoding stage, learning inter-node association strengths end-to-end through a relation encoder, and leveraging graph attention to achieve bidirectional cross-object context propagation.

## Method

### Ethics approval and consent to participate

Not applicable.

### Problem definition

Given a sports scene image $I\in {\mathbb{R}}^{H\times W\times 3}$, the object detection task aims to predict a set of targets $𝒴=\{(c_{i},{𝐛}_{i}){\}}_{i=1}^{N}$, where $c_{i}\in \{1,...,N_{c}\}$ is the category label and ${𝐛}_{i}=(c_{x},c_{y},w,h)\in [0,1{]}^{4}$ is the normalized bounding box coordinates. To unify annotation granularity across datasets, we map players, goalkeepers, referees, assistant referees, and staff in SoccerNet-Tracking to the Player class and retain the ball as the Ball class; SportsMOT directly uses its original category annotations; Badminton-Players-Detection contains Player and Shuttlecock categories.

Compared with general natural scenes, target distribution in sports scenes exhibits two distinctive domain characteristics. First, the field region provides explicit spatial boundary constraints for target distribution, with all detection targets appearing within the field, while off-field regions (spectator stands, billboards, etc.) that occupy a large portion of the frame contain no targets yet frequently trigger false detections due to the visual similarity between spectators and athletes. Second, objects of different categories such as athletes, balls, and referees form stable spatial co-occurrence patterns governed by game rules, for example the football always remains within players’ reachable range and referees maintain specific distances from contested areas, yet existing detectors predict each object independently and cannot leverage these cross-category relational cues. SportRGDET explicitly models and injects these two types of domain priors into the detection pipeline through the SPGM and RREM modules, respectively.

### Architecture overview

The overall architecture of SportRGDET is shown in Fig 2, built upon Deformable DETR [5]. Given an input image *I*, the framework consists of the following components: (1) a ResNet-50 backbone with FPN extracts multi-scale features, flattened into a token sequence $𝐗\in {\mathbb{R}}^{L\times d}$; (2) the Scene Prior Guided Module (SPGM) estimates a spatial confidence field ϕ from 𝐗 and injects it into the deformable self-attention layers of the Transformer encoder for spatial modulation, reallocating attention resources toward within-field regions; (3) the Transformer decoder extracts candidate objects from encoded features through object queries and cross-attention; (4) the Relation Reasoning Enhancement Module (RREM) organizes candidate objects into a dynamic relation graph, propagating cross-category contextual information between nodes via graph attention to refine predictions of difficult targets; (5) the prediction head fuses relation reasoning results in a residual manner to output final detections. The two core modules operate at the feature encoding and object decoding stages respectively, jointly improving detection performance from the complementary dimensions of spatial focusing and relation reasoning. The following subsections describe the design of each component in detail.

**Fig 2:**
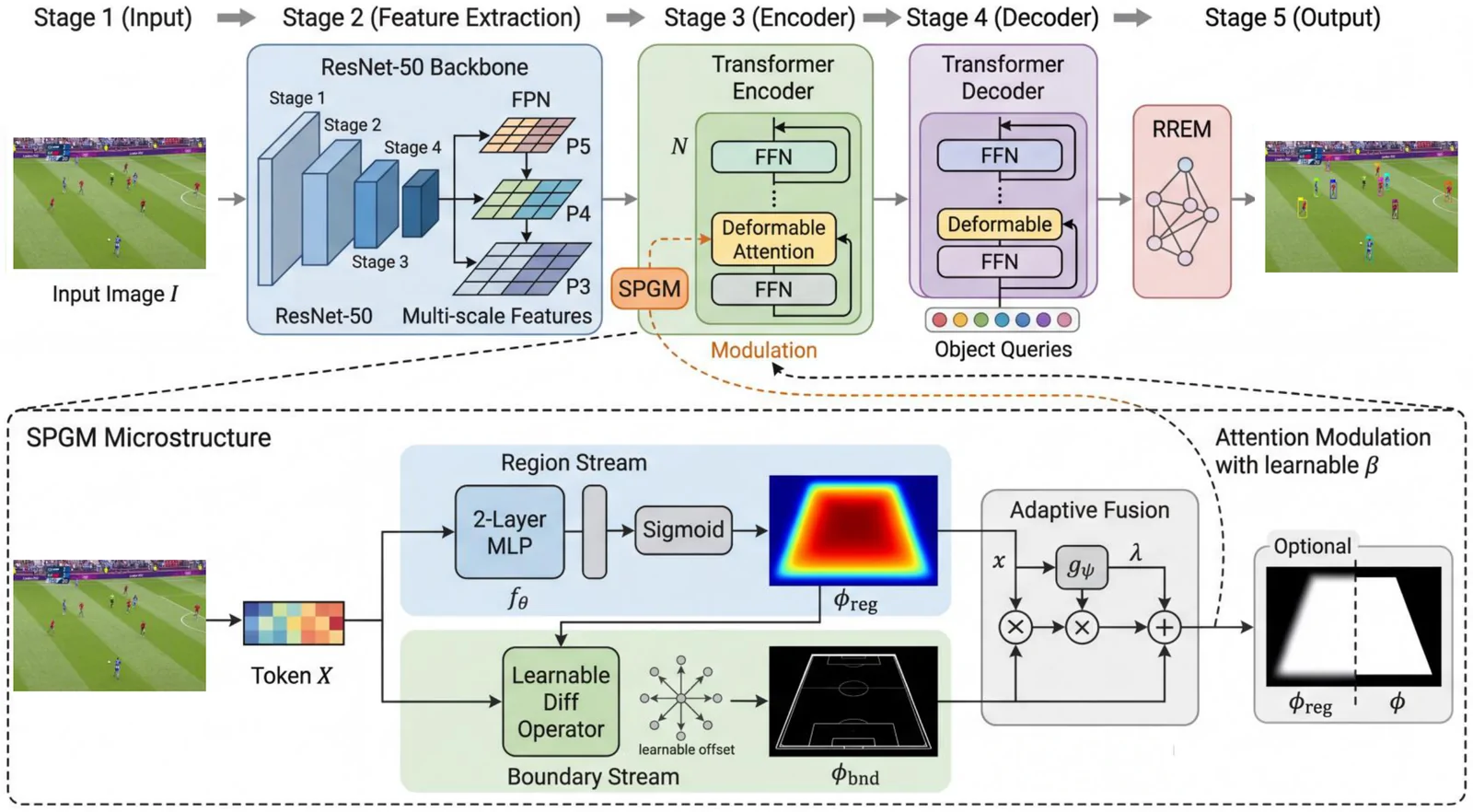
Overall architecture of SportRGDET and SPGM module details. The upper part illustrates the complete pipeline from input image to detection output: Stage 1 (Input) → Stage 2 (ResNet-50 + FPN multi-scale feature extraction) → Stage 3 (SPGM-modulated Transformer encoder with deformable self-attention) → Stage 4 (Transformer decoder with cross-attention to encoder features) → Stage 5 (RREM relation reasoning enhancement + output). The lower part shows the SPGM microstructure: the token sequence 𝐗 undergoes dual-stream estimation via the region stream ($f_{\theta }\to \phi_{\text{reg}}$) and boundary stream (learnable differential operator $\to \phi_{\text{bnd}}$), followed by adaptive fusion ($g_{\psi }\to \lambda$) to generate the final confidence field ϕ, which progressively modulates deformable self-attention with learnable strength β.

### Multi-scale feature extraction

SportRGDET requires both global field layout information and local target detail features, thus we construct a multi-scale feature extraction pipeline. For an input image *I*, the feature extraction process can be uniformly expressed as:


$$\begin{array}{c} \left\{P_{l}{\}}_{l=3}^{5}={ℱ}_{\text{enc}}(I;\Theta_{\text{enc}}\right) \end{array}$$ (1)

where ${ℱ}_{\text{enc}}(\cdot ;\Theta_{\text{enc}})$ comprises the backbone network and multi-scale fusion stages. The backbone adopts an ImageNet-pretrained ResNet-50, outputting hierarchical features $\left\{C_{3},C_{4},C_{5}\right\}$ from three stages with strides of 8, 16, and 32, respectively. These are subsequently fused through a Feature Pyramid Network (FPN) for cross-scale integration, with 1×1 convolutions unifying channel dimensions to *d* = 256 and a top-down pathway progressively fusing high-level semantics with low-level spatial details, producing multi-scale features $P_{l}\in {\mathbb{R}}^{d\times H_{l}\times W_{l}}$. To incorporate multi-scale features into a unified Transformer processing framework, we flatten and concatenate all scales into a token sequence $𝐗=\{{𝐱}_{i}{\}}_{i=1}^{L}\in {\mathbb{R}}^{L\times d}$, where $L=\sum_{l=3}^{5}H_{l}W_{l}$, with each token ${𝐱}_{i}$ augmented with sinusoidal position encodings and learnable scale embeddings to preserve spatial coordinates ${𝐩}_{i}\in [0,1{]}^{2}$ and scale information. 𝐗 simultaneously serves as input to both the SPGM confidence field estimation and the Transformer encoder attention computation.

### Scene prior guided module (SPGM)

Generic detectors uniformly allocate attention across all spatial positions, but sports scenes possess a strong prior: detection targets only appear within the field region $\Omega_{\text{field}}\subset {\mathbb{R}}^{2}$, while off-field regions that occupy a large portion of the frame contain no targets yet introduce false detections. Starting from the token sequence 𝐗, SPGM formalizes the field spatial prior as a spatial confidence field $\phi :[0,1{]}^{2}\to [0,1]$, whose physical meaning is the prior probability that an effective detection target exists at coordinate 𝐩. The goal of this module is to estimate ϕ from 𝐗 and inject it as a soft constraint into the encoder’s deformable self-attention mechanism, aligning the spatial distribution of attention responses with the field structure. We stress that this modulation does *not* prune tokens or reduce theoretical FLOPs; it *regularizes* where deformable sampling weight is concentrated. The resulting accuracy and overhead trade-off is measured directly in Table 8.

#### Dual-stream confidence field estimation.

An ideal confidence field ϕ should simultaneously satisfy two properties: (i) values close to 1 inside the field and close to 0 in off-field regions (region discriminability); (ii) a steep transition rather than smooth decay at field boundaries (boundary sharpness). A single semantic segmentation network cannot guarantee both properties simultaneously, as segmentation networks tend to produce smooth probability outputs, resulting in insufficient modulation effects in the boundary transition zone. To this end, we decompose ϕ into two complementary signals, the region stream $\phi_{\text{reg}}$ and the boundary stream $\phi_{\text{bnd}}$, and estimate them jointly.

The region stream predicts the field membership probability for each spatial position from the token sequence 𝐗. For token ${𝐱}_{i}$ and its corresponding spatial coordinate ${𝐩}_{i}$, the region stream outputs a scalar confidence through a lightweight projection $f_{\theta }$:


$$\begin{array}{c} \phi_{\text{reg}}({𝐩}_{i})=\sigma (f_{\theta }({𝐱}_{i}))\in [0,1] \end{array}$$ (2)

where $f_{\theta }$ is a two-layer MLP and σ is the Sigmoid function. $\phi_{\text{reg}}$ approaches 1 inside the field and 0 in off-field regions, supervised by an auxiliary binary cross-entropy loss ${ℒ}_{\text{seg}}$. However, the output of $\phi_{\text{reg}}$ transitions smoothly near field boundaries, resulting in insufficiently sharp modulation. The boundary stream explicitly estimates the spatial gradient of $\phi_{\text{reg}}$ through a learnable differential operator to capture boundary locations. Specifically, centered at ${𝐩}_{i}$, the region stream outputs are sampled at $K_{g}$ learnable offset positions and directionally weighted differences are computed:


$$\begin{array}{c} \phi_{\text{bnd}}({𝐩}_{i})={\left\|\sum_{k=1}^{K_{g}}\eta_{k}\cdot [\phi_{\text{reg}}({𝐩}_{i}+\delta_{k})-\phi_{\text{reg}}({𝐩}_{i})]\cdot \frac{\delta_{k}}{\left\|\delta_{k}\right\|}\right\|}_{2} \end{array}$$ (3)

where the offsets $\left\{\delta_{k}\right\}$ and weights $\left\{\eta_{k}\right\}$ are all learnable parameters, with $K_{g}=8$. Unlike fixed Sobel operators, the learnable differential operator can adaptively adjust its sensitivity to gradient changes along different directions and distances. For example, for a rectangular football pitch, the network can learn to assign larger weights along the long-edge direction to precisely capture horizontal boundaries. $\phi_{\text{bnd}}$ takes large values at field boundaries and approaches zero in the flat interior and exterior regions.

#### Adaptive dual-stream fusion.

The region stream provides region discrimination of “where the field is,” while the boundary stream provides precise localization of “where the boundary is.” The optimal fusion ratio between the two should depend on local context. In field interior regions far from boundaries, the high confidence of the region stream is sufficient; in the boundary transition zone, the boundary stream’s contribution needs to be enhanced to achieve sharp cutoffs. We adaptively predict fusion coefficients $\lambda_{i}=\sigma (g_{\psi }({𝐱}_{i}))$ from each token’s features to synthesize the final confidence field:


$$\begin{array}{c} \phi ({𝐩}_{i})=\lambda_{i}\cdot \phi_{\text{reg}}({𝐩}_{i})+(1-\lambda_{i})\cdot \phi_{\text{reg}}({𝐩}_{i})\cdot (1-\phi_{\text{bnd}}({𝐩}_{i})) \end{array}$$ (4)

When $\lambda_{i}\to 1$ (far from the boundary), ϕ degenerates to the pure region stream output; when $\lambda_{i}\to 0$ (near the boundary), ϕ is jointly determined by the region probability multiplied by the boundary suppression factor $\left(1-\phi_{\text{bnd}}\right)$, producing a steeper confidence decay at boundaries.

#### Progressive attention modulation.

After obtaining the confidence field ϕ, we inject it into the deformable self-attention mechanism inside each layer of the Transformer encoder (following the Deformable DETR encoder and decoder split, in which encoder layers use deformable self-attention over image tokens, while cross-attention to encoder features occurs inside the decoder [5]). Unlike hard cropping or feature scaling, we perform modulation at the deformable sampling-weight level. Let the un-normalized deformable attention weight from token *i* to its *j*-th sampling location in the *n*-th encoder layer be $\kappa^{\left(n\right)}({𝐱}_{i},{𝐱}_{j})$; we define the prior-modulated deformable weight as:


$$\begin{array}{c} {\tilde{\kappa }}^{\left(n\right)}({𝐱}_{i},{𝐱}_{j})=\kappa^{\left(n\right)}({𝐱}_{i},{𝐱}_{j})\cdot [(1-\beta^{\left(n\right)})+\beta^{\left(n\right)}\cdot \phi ({𝐩}_{j})] \end{array}$$ (5)

where $\beta^{\left(n\right)}\in [0,1]$ is a per-layer learnable modulation strength parameter. This design achieves progressive regularization: in shallow layers, $\beta^{\left(n\right)}$ is small, allowing the encoder to freely model low-level textures and structures across the entire image; as the layer depth increases, $\beta^{\left(n\right)}$ gradually grows, continuously reducing the weight of off-field tokens in attention aggregation. The update of token *i* in the *n*-th encoder layer is:


$$\begin{array}{c} {\hat{𝐱}}_{i}^{\left(n\right)}={𝐱}_{i}^{\left(n\right)}+\sum_{j\in {𝒩}_{i}}\frac{{\tilde{\kappa }}^{\left(n\right)}({𝐱}_{i},{𝐱}_{j})}{\sum_{j^{\prime }}{\tilde{\kappa }}^{\left(n\right)}({𝐱}_{i},{𝐱}_{j^{\prime }})}\cdot W_{v}^{\left(n\right)}{𝐱}_{j}^{\left(n\right)} \end{array}$$ (6)

where ${𝒩}_{i}$ is the set of deformable sampling locations for token *i*. The field prior ϕ performs a spatial re-weighting of the deformable sampling weights, redistributing attention resources, originally distributed uniformly across on-field and off-field regions, toward the field area, rather than discarding off-field features or reducing the number of sampling operations. Residual connections and corrections from subsequent layers provide a fault-tolerance mechanism for local prediction errors in the confidence field. After *N* encoder layers, the modulated feature sequence $\hat{𝐗}=\{{\hat{𝐱}}_{i}{\}}_{i=1}^{L}$ is output and fed into the Transformer decoder to extract candidate targets.

#### Relation reasoning enhancement module (RREM).

The Transformer decoder interacts with the encoder output $\hat{𝐗}$ through $N_{q}$ learnable object queries via cross-attention, generating for each query an instance embedding ${𝐞}_{i}\in {\mathbb{R}}^{d}$, preliminary bounding-box logits ${𝐮}_{i}^{\text{dec}}\in {\mathbb{R}}^{4}$ (with the decoded box ${𝐛}_{i}^{\text{dec}}=\sigma ({𝐮}_{i}^{\text{dec}})$), and class logits ${𝐳}_{i}^{\text{dec}}\in {\mathbb{R}}^{N_{c}}$ (with class probabilities ${𝐩}_{i}^{\text{cls,dec}}=Softmax({𝐳}_{i}^{\text{dec}})$). However, these predictions are generated independently per target, completely discarding the spatial co-occurrence relationships between targets of different categories that are constrained by game rules in sports scenes. RREM (illustrated in Fig 3) organizes the $N_{q}$ candidate targets into a dynamic graph structure, adaptively learning inter-node association strengths through a relation encoder and bidirectionally propagating contextual information between nodes via multi-head graph attention.

**Fig 3:**
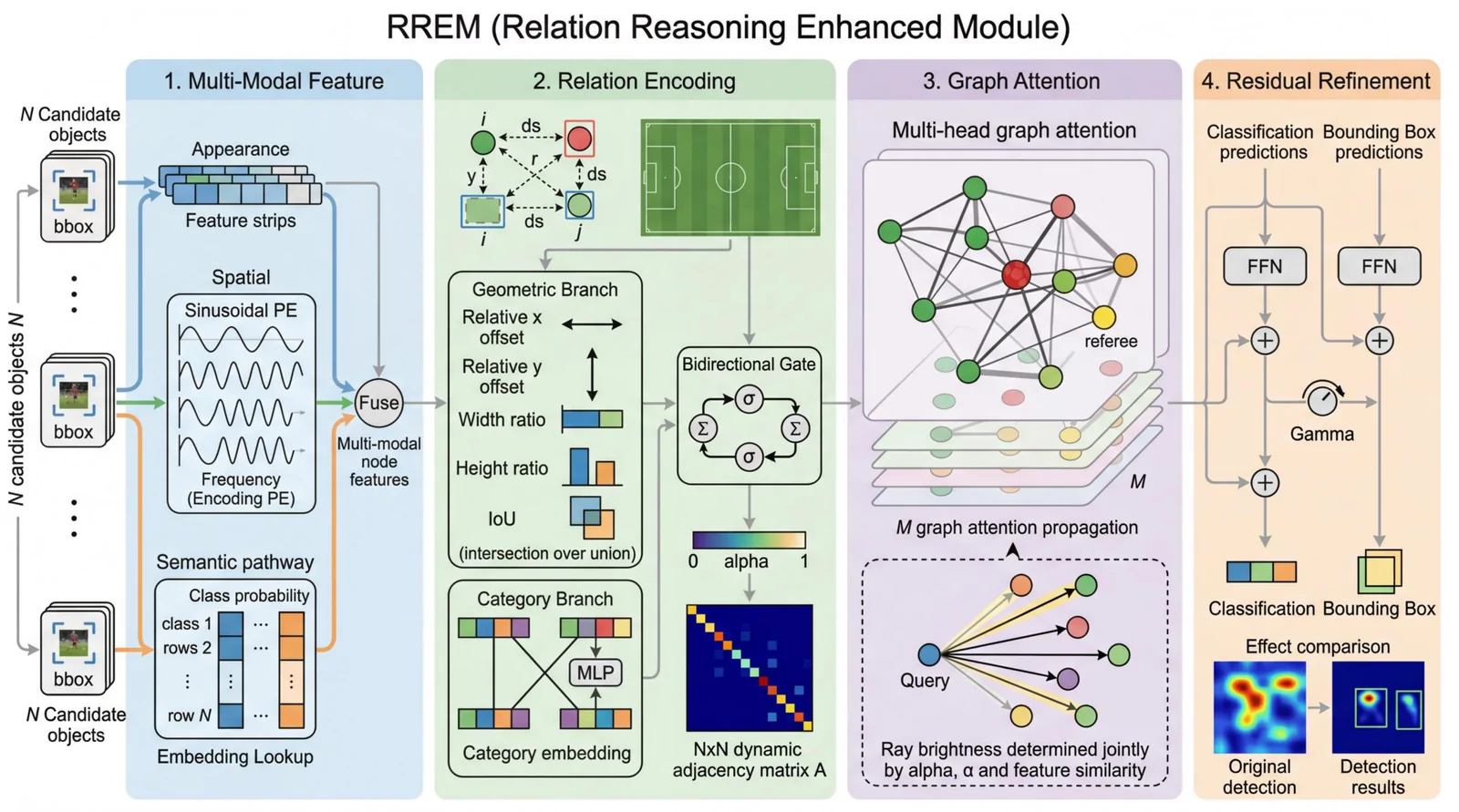
Detailed structure of the RREM module. It contains four sub-components: (1) Multi-modal node feature construction, fusing candidate objects’ appearance embeddings, sinusoidal position encodings, and category semantic embeddings into unified node representations; (2) Relation encoding, where the geometric branch extracts normalized relative spatial features (offsets, scale ratios, IoU) and the category branch generates category representations through embedding lookup, fused via bidirectional gating to predict association strengths α forming the N×N dynamic adjacency matrix; (3) Multi-head graph attention propagation, where α serves as structural priors jointly determining attention weights with feature similarity across *M* layers of propagation to fuse cross-object context; (4) Residual refinement prediction head operating in logit / inverse-sigmoid space, adding relation reasoning corrections to the decoder’s original prediction logits in a residual manner with learnable scaling factors γ.

#### Multi-modal node feature construction.

Based on the decoder outputs ${𝐞}_{i}$, ${𝐛}_{i}^{\text{dec}}$, and ${𝐩}_{i}^{\text{cls,dec}}$, we construct multi-modal node features that fuse appearance, spatial, and semantic information for each candidate target. Spatial positions are mapped to a high-dimensional space through multi-frequency sinusoidal encoding $\text{PE}({𝐛}_{i}^{\text{dec}})\in {\mathbb{R}}^{d_{p}}$. Category semantics are generated via soft weighting using a learnable category embedding matrix ${𝐄}_{c}\in {\mathbb{R}}^{N_{c}\times d_{c}}$:


$$\begin{array}{c} {𝐬}_{i}=({𝐩}_{i}^{\text{cls,dec}}{)}^{⊤}{𝐄}_{c}\in {\mathbb{R}}^{d_{c}} \end{array}$$ (7)

where ${𝐬}_{i}$ is the category semantic embedding, obtained by soft-weighting the embedding matrix with class probabilities ${𝐩}_{i}^{\text{cls,dec}}$. The appearance embedding ${𝐞}_{i}$, spatial position encoding $\text{PE}({𝐛}_{i}^{\text{dec}})$, and category semantic embedding ${𝐬}_{i}$ from three modalities are concatenated and fused through linear projection into a unified node representation:


$$\begin{array}{c} {𝐡}_{i}^{\left(0\right)}=\text{LN}(W_{h}[{𝐞}_{i};\text{PE}({𝐛}_{i}^{\text{dec}});{𝐬}_{i}])\in {\mathbb{R}}^{d_{n}} \end{array}$$ (8)

#### Category-spatial joint relation encoder.

The relation encoder predicts an association strength $\alpha_{ij}\in [0,1]$ for each pair of candidate targets (*i*, *j*), evaluating from two dimensions: category semantic compatibility and spatial relationship plausibility. For candidate targets *i* and *j* with bounding boxes ${𝐛}_{i}^{\text{dec}}=(c_{x}^{i},c_{y}^{i},w_{i},h_{i})$ and ${𝐛}_{j}^{\text{dec}}=(c_{x}^{j},c_{y}^{j},w_{j},h_{j})$, where $\left(c_{x},c_{y}\right)$ denotes the box center coordinates and (*w*, *h*) the box width and height, we adopt a geometric feature representation normalized relative to the subject target to ensure scale invariance of spatial encoding:


$$\begin{array}{c} \Delta {𝐠}_{ij}=\left(\frac{c_{x}^{j}-c_{x}^{i}}{w_{i}},\frac{c_{y}^{j}-c_{y}^{i}}{h_{i}},\log\frac{w_{j}}{w_{i}},\log\frac{h_{j}}{h_{i}},\text{IoU}({𝐛}_{i}^{\text{dec}},{𝐛}_{j}^{\text{dec}})\right) \end{array}$$ (9)

The geometric encoding ${𝐫}_{ij}^{\text{geo}}$ and category compatibility encoding ${𝐫}_{ij}^{\text{cls}}$ are extracted by independent MLPs from $\Delta {𝐠}_{ij}$ and the category embedding pair $\left({𝐬}_{i},{𝐬}_{j}\right)$, respectively, and then fused through a bidirectional gating mechanism to predict association strength:


$$\begin{array}{c} {𝐟}_{ij}={𝐫}_{ij}^{\text{geo}}⊙\sigma (W_{g}{𝐫}_{ij}^{\text{cls}})+{𝐫}_{ij}^{\text{cls}}⊙\sigma (W_{g}^{\prime }{𝐫}_{ij}^{\text{geo}}) \end{array}$$ (10)

$$\begin{array}{c} \alpha_{ij}=\sigma ({𝐰}_{\alpha }^{⊤}{𝐟}_{ij}+b_{\alpha }) \end{array}$$ (11)

The gating mechanism enables mutual modulation between the two encodings: when category compatibility is high (e.g., player and ball), the gating signal enhances the role of geometric encoding; when category compatibility is low, the gating signal suppresses geometric encoding to avoid spurious associations caused solely by spatial proximity. This design instantiates the calibrated (rather than unconditional) alignment principle of Fang et al. [1]. The set $\left\{\alpha_{ij}\right\}$ forms a dynamic adjacency matrix $𝐀\in {\mathbb{R}}^{N_{q}\times N_{q}}$, learned entirely end-to-end by the network without manually defined graph topology.

#### Multi-head graph attention information propagation.

Based on the dynamic relational graph, we propagate contextual information between nodes through multi-head graph attention. Unlike standard GAT, the association strength $\alpha_{ij}$ predicted by the relation encoder is embedded as a structural prior into the attention computation. In the *h*-th head of the *m*-th layer, the attention weight from node *i* to node *j* is:


$$\begin{array}{c} {\hat{\alpha }}_{ij}^{\left(m,h\right)}=\frac{\alpha_{ij}\cdot \exp\left(\frac{\left({𝐡}_{i}^{\left(m\right)}W_{q}^{\left(m,h\right)}{)}^{⊤}({𝐡}_{j}^{\left(m\right)}W_{k}^{\left(m,h\right)}\right)}{\sqrt{d_{n}/N_{h}}}\right)}{\sum_{l=1}^{N_{q}}\alpha_{il}\cdot \exp\left(\frac{\left({𝐡}_{i}^{\left(m\right)}W_{q}^{\left(m,h\right)}{)}^{⊤}({𝐡}_{l}^{\left(m\right)}W_{k}^{\left(m,h\right)}\right)}{\sqrt{d_{n}/N_{h}}}\right)} \end{array}$$ (12)

When $\alpha_{ij}\approx 0$, regardless of how high the feature similarity is, node *j*’s contribution is suppressed to near zero, effectively blocking information leakage between unrelated targets. The outputs of all heads are concatenated and node features are updated in a residual manner:


$$\begin{array}{c} {𝐡}_{i}^{\left(m+1\right)}=\text{LN}\left({𝐡}_{i}^{\left(m\right)}+W_{o}^{\left(m\right)}\overset{N_{h}}{\underset{h=1}{⨁}}\sum_{j=1}^{N_{q}}{\hat{\alpha }}_{ij}^{\left(m,h\right)}\cdot W_{v}^{\left(m,h\right)}{𝐡}_{j}^{\left(m\right)}\right) \end{array}$$ (13)

A feed-forward network follows each graph attention layer to enhance nonlinear transformation capacity. After *M* layers of propagation, the node features ${𝐡}_{i}^{\left(M\right)}$ have incorporated contextual information from associated targets.

#### Relation-enhanced prediction head.

To make the residual correction well-defined and to guarantee that RREM approximates an *exact* identity mapping at initialization, we operate in *logit / inverse-sigmoid* space rather than directly on probability and normalized-box outputs. Let ${𝐳}_{i}^{\text{dec}}\in {\mathbb{R}}^{N_{c}}$ denote the decoder’s classification logits (so that ${𝐩}_{i}^{\text{cls,dec}}=Softmax({𝐳}_{i}^{\text{dec}})$), and let ${𝐮}_{i}^{\text{dec}}=\sigma^{-1}({𝐛}_{i}^{\text{dec}})\in {\mathbb{R}}^{4}$ denote the inverse-sigmoid (i.e., logit) representation of the decoded normalized bounding box ${𝐛}_{i}^{\text{dec}}$, following the iterative refinement convention of Deformable DETR [5] and DAB-DETR [6]. The enhanced node features pass through independent classification and regression branches to correct the decoder’s preliminary predictions in a residual manner in logit space:


$$\begin{array}{c} {\hat{𝐳}}_{i}={𝐳}_{i}^{\text{dec}}+\gamma_{c}\cdot {\text{FFN}}_{\text{cls}}({𝐡}_{i}^{\left(M\right)}),\;{\hat{𝐮}}_{i}={𝐮}_{i}^{\text{dec}}+\gamma_{b}\cdot {\text{FFN}}_{\text{box}}({𝐡}_{i}^{\left(M\right)}) \end{array}$$ (14)

$$\begin{array}{c} {\hat{p}}_{i}^{\text{cls}}=Softmax({\hat{𝐳}}_{i}),\;{\hat{𝐛}}_{i}=\sigma ({\hat{𝐮}}_{i}) \end{array}$$ (15)

Under this formulation, when the learnable scaling factors $\gamma_{c}$ and $\gamma_{b}$ equal zero, we have ${\hat{𝐳}}_{i}={𝐳}_{i}^{\text{dec}}$ and ${\hat{𝐮}}_{i}={𝐮}_{i}^{\text{dec}}$, so that ${\hat{p}}_{i}^{\text{cls}}={𝐩}_{i}^{\text{cls,dec}}$ and ${\hat{𝐛}}_{i}=\sigma ({𝐮}_{i}^{\text{dec}})={𝐛}_{i}^{\text{dec}}$. RREM therefore reduces to an *exact* identity mapping at initialization, without interfering with the convergence of the base detector. $\gamma_{c}$ and $\gamma_{b}$ are initialized to small values (0.1) and jointly optimized with the rest of the network.

### Detection head and label assignment

The Transformer decoder uses $N_{q}=300$ learnable object queries, interacting with encoder features $\hat{𝐗}$ through 6 layers of cross-attention. Each decoder layer contains self-attention (inter-query interaction), cross-attention (query-to-encoded-feature interaction), and a feed-forward network, progressively refining the representation of candidate targets layer by layer. Label assignment adopts the Hungarian matching algorithm to find the optimal one-to-one correspondence $\hat{\pi }$ between the prediction set and the ground-truth target set:


$$\begin{array}{c} \hat{\pi }=\text{arg}\min_{\pi \in \Pi_{N_{q}}}\sum_{i=1}^{N}{𝒞}_{\text{match}}({𝒴}_{i},{\hat{𝒴}}_{\pi (i)}) \end{array}$$ (16)

where $\Pi_{N_{q}}$ is the set of all permutations of $N_{q}$ elements and *N* is the number of ground-truth targets. The matching cost ${𝒞}_{\text{match}}$ is a weighted sum of classification cost, L1 distance cost, and GIoU cost:


$$\begin{array}{c} {𝒞}_{\text{match}}=\lambda_{\text{cls}}{𝒞}_{\text{cls}}+\lambda_{\text{L1}}\|{𝐛}_{i}^{\text{gt}}-{\hat{𝐛}}_{\pi (i)}{\|}_{1}+\lambda_{\text{GIoU}}{𝒞}_{\text{GIoU}}({𝐛}_{i}^{\text{gt}},{\hat{𝐛}}_{\pi (i)}) \end{array}$$ (17)

where ${𝒞}_{\text{cls}}$ is the cost term based on classification probabilities and ${𝐛}_{i}^{\text{gt}}$ is the bounding box of the *i*-th ground-truth target. Consistent with the original DETR / Deformable DETR formulation [4,5], the Hungarian matcher expands the ground-truth set to size $N_{q}$ by padding with the *no-object* label ∅, so that every query is matched to either a ground-truth target or ∅. Queries matched to ∅ are supervised only by the classification loss with target class ∅; the box regression loss is not applied to them. The equations above are the abbreviated versions written for matched queries, and ${ℒ}_{\text{det}}$ in Eq 19 below likewise omits the no-object classification term for brevity; both terms follow the standard DETR implementation. The decoder employs auxiliary decoding losses, applying classification and regression supervision at the output of every layer to accelerate convergence.

### Loss function

The overall training loss consists of the base detection loss, the field segmentation auxiliary loss, and the relational consistency loss:


$$\begin{array}{c} {ℒ}_{\text{total}}={ℒ}_{\text{det}}+\lambda_{\text{seg}}{ℒ}_{\text{seg}}+\lambda_{\text{rel}}{ℒ}_{\text{rel}} \end{array}$$ (18)

During training, Hungarian matching is first performed using the matching cost ${𝒞}_{\text{match}}$ (Eq 16) to solve for the optimal one-to-one correspondence $\hat{\pi }$ between predictions and ground-truth targets (Eq 15). This matching process does not participate in gradient computation and is only used to determine “which prediction corresponds to which ground-truth target.” Subsequently, based on the matching result $\hat{\pi }$, ${ℒ}_{\text{det}}$ applies classification and localization supervision to each prediction matched to a ground-truth target (with an additional no-object classification term on unmatched queries as noted above):


$$\begin{array}{c} {ℒ}_{\text{det}}=\sum_{i=1}^{N}\left[\lambda_{\text{cls}}{ℒ}_{\text{focal}}(c_{i}^{\text{gt}},{\hat{p}}_{\hat{\pi }(i)}^{\text{cls}})+\lambda_{\text{L1}}\|{𝐛}_{i}^{\text{gt}}-{\hat{𝐛}}_{\hat{\pi }(i)}{\|}_{1}+\lambda_{\text{GIoU}}{ℒ}_{\text{GIoU}}({𝐛}_{i}^{\text{gt}},{\hat{𝐛}}_{\hat{\pi }(i)})\right] \end{array}$$ (19)

where ${ℒ}_{\text{focal}}$ is Focal Loss ($\alpha_{t}=0.25$, γ=2), $\lambda_{\text{cls}}=2.0$, $\lambda_{\text{L1}}=5.0$, $\lambda_{\text{GIoU}}=2.0$. ${ℒ}_{\text{seg}}$ is the auxiliary binary cross-entropy loss for the region stream $\phi_{\text{reg}}$, supervised by the field mask ground truth $y_{i}\in \{0,1\}$:


$$\begin{array}{c} {ℒ}_{\text{seg}}=-\frac{1}{L}\sum_{i=1}^{L}\left[y_{i}\log\phi_{\text{reg}}({𝐩}_{i})+(1-y_{i})\log(1-\phi_{\text{reg}}({𝐩}_{i}))\right] \end{array}$$ (20)

This loss is only used during the training phase; at inference time, the field segmentation branch adds negligible computational overhead. ${ℒ}_{\text{rel}}$ guides the relation encoder to learn meaningful association patterns, constructing relational pseudo-labels for matched candidate target pairs based on the matching result $\hat{\pi }$:


$$\begin{array}{c} y_{ij}^{\text{rel}}=\exp\left(-\frac{\|{𝐜}_{i}^{\text{gt}}-{𝐜}_{j}^{\text{gt}}{\|}_{2}^{2}}{2\tau_{r}^{2}}\right)\cdot 1[c_{i}^{\text{gt}}\neq c_{j}^{\text{gt}}\vee \text{IoU}({𝐛}_{i}^{\text{gt}},{𝐛}_{j}^{\text{gt}})>0] \end{array}$$ (21)

$$\begin{array}{c} {ℒ}_{\text{rel}}=-\frac{1}{|ℳ{|}^{2}}\sum_{(i,j)\in {ℳ}^{2}}[y_{ij}^{\text{rel}}\log\alpha_{ij}+(1-y_{ij}^{\text{rel}})\log(1-\alpha_{ij})] \end{array}$$ (22)

where ${𝐜}_{i}^{\text{gt}}$ is the ground-truth box center coordinate, $\tau_{r}$ is the distance temperature parameter, and ℳ is the set of candidate indices matched to ground-truth targets. In experiments, we set $\lambda_{\text{seg}}=1.0$, $\lambda_{\text{rel}}=0.5$, $\tau_{r}=0.3$, graph attention layers *M* = 3, and number of heads $N_{h}=8$.

## Experiments

### Datasets

We evaluate SportRGDET on three datasets: SportsMOT, Badminton-Players-Detection, and SoccerNet-Tracking, covering multi-sport team scenarios, racket sport close-range scenarios, and large-scale soccer broadcast scenarios, respectively. To unify the task definition, we convert the tracking annotations of SportsMOT and SoccerNet-Tracking into detection annotations: per-frame bounding boxes are retained while track IDs are removed, and the data is used solely for object detection training and evaluation.

**SportsMOT.** This dataset contains three types of sports scenes: basketball, volleyball, and football, comprising 240 video clips, 150,379 frames, 1,629,490 annotated bounding boxes, and 3,401 tracks. The data is split into train/val/test = 45/45/150. The video resolution is 1280×720 at 25 FPS. We train and validate on its official train/val split and report results under the detection setting.

**Badminton-Players-Detection.** This is a publicly available badminton detection dataset containing 1,022 images with two categories: player and shuttlecock, split into train/valid/test = 819/139/64. The dataset contains a total of 3,197 annotated bounding boxes, of which 2,370 are player and 827 are shuttlecock; all images are resized to 1280×1280. The main experiments focus on player detection, with shuttlecock results reported as supplementary.

**SoccerNet-Tracking.** SoccerNet-Tracking is currently the largest soccer video multi-object tracking benchmark, containing 200 video clips of 30 seconds each sourced from the main broadcast camera of 12 professional soccer matches, plus one complete 45-minute half. The video resolution is 1920×1080 at 25 FPS. All visible targets on the pitch are densely annotated, including players from both teams, goalkeepers, referees, assistant referees, and the ball. The dataset is officially split into training (57 sequences), test (49 sequences), and challenge (58 sequences) sets. We use the official training and test sets for training and evaluation under the detection setting.

### Field-mask annotation protocol

Because SPGM uses field masks as auxiliary supervision, we describe the mask generation and correction protocol in detail here in the interest of reproducibility and to enable readers to reproduce or audit our supervision budget.

**Automatic mask generation.** For SoccerNet-Tracking and SportsMOT we generate initial field masks using a DeepLabV3+ [24] model with a ResNet-101 backbone. The segmentation model was itself trained on approximately 2,500 field-region masks from public sports segmentation resources (SoccerNet field-plane annotations [9], sports court subsets of ADE20K [25] and Mapillary [26]), *disjoint* from the SoccerNet-Tracking and SportsMOT sequences used in this paper. No frame from the detection test sets was ever included in the DeepLabV3 + training data. For the Badminton-Players-Detection dataset, whose court is a regular rectangle with fixed geometry, we directly use axis-aligned rectangular masks derived from the four visible corner court lines; no learned segmenter is used.

**Manual review budget.** Two graduate students with sports domain background inspected the automatically generated masks. Manual review was carried out on a stratified subsample of the *training splits only*: 812 frames sampled uniformly across the 57 SoccerNet-Tracking training sequences (approximately 14 frames per sequence) and 630 frames sampled from the 45 SportsMOT training sequences (14 per sequence). No frame from the validation or test splits of any dataset was ever inspected, annotated, or corrected. Corrections consisted of touching-up polygon vertices along the field boundary using CVAT; blanket redraws were performed only when the automatic mask exhibited catastrophic drift (occurring on 4.8% of reviewed frames). Median annotation time per frame was 43 seconds. On the reviewed frames, mean field-mask IoU relative to consensus ground truth rose from 82.5 (raw DeepLabV3+) to 94.2, and mean F1 from 89.4 to 97.1.

**Mask availability.** All 1,442 manually reviewed masks together with the automatic masks, the CVAT project files, and the annotation guideline document will be released together with the code upon publication.

### Implementation details

We implement SportRGDET based on Deformable DETR, using an ImageNet-pretrained ResNet-50 as the backbone, which outputs three-scale features $\left\{C_{3},C_{4},C_{5}\right\}$ fused through FPN into *d* = 256-dimensional multi-scale representations, flattened into the token sequence 𝐗. The detection head follows the Deformable DETR configuration (6-layer decoder, $N_{q}=300$ queries) with Hungarian matching for label assignment.

In SPGM, the region stream projection $f_{\theta }$ uses a two-layer MLP (256→64→1), the learnable differential operator in the boundary stream has $K_{g}=8$ sampling points with offsets $\left\{\delta_{k}\right\}$ initialized as a uniform circular distribution centered at the origin, and the fusion coefficient projection $g_{\psi }$ is a single linear layer. In the progressive attention modulation, the Transformer encoder has 6 layers with per-layer modulation strength $\beta^{\left(n\right)}$ initialized to 0.1 and adaptively adjusted during training, and each deformable self-attention head uses $K_{s}=4$ sampling points. The field-mask annotation protocol used to supervise $\phi_{\text{reg}}$ is documented in the previous subsection. In RREM, the category embedding matrix dimension is $d_{c}=64$, node feature dimension $d_{n}=256$, and spatial position encoding frequency order $L_{f}=16$ ($d_{p}=128$). The geometric and category encoding intermediate dimension in the relation encoder is $d_{r}=64$, and multi-head graph attention uses *M* = 3 layers with $N_{h}=8$ heads. The residual prediction head scaling factors $\gamma_{c}$ and $\gamma_{b}$ are initialized to 0.1, ensuring RREM approximates an exact identity mapping in logit space during early training.

Training uses the AdamW optimizer with a backbone learning rate of $1\times 10^{-5}$ and $1\times 10^{-4}$ for other modules, weight decay of $1\times 10^{-4}$, for 150 epochs (10-epoch linear warmup, cosine annealing schedule). The total batch size is 16 (4 RTX 4090 GPUs, 4 per GPU). Input resolution is set to 1920×1080 for SoccerNet-Tracking, 1280×720 for SportsMOT, and 1280×1280 for Badminton-Players-Detection. Data augmentation includes random horizontal flipping, 0.8~1.2× random scaling, color jittering, and random cropping. Loss weights are set to $\lambda_{\text{cls}}=2.0$, $\lambda_{\text{L1}}=5.0$, $\lambda_{\text{GIoU}}=2.0$, $\lambda_{\text{seg}}=1.0$, $\lambda_{\text{rel}}=0.5$, with the distance temperature parameter for relation pseudo-labels $\tau_{r}=0.3$. At test time, no multi-scale testing or model ensembling is used, with a confidence threshold of 0.3. We report mAP@0.5 and mAP@0.75 as primary metrics along with per-class detection accuracy, and measure inference speed in FPS on a single RTX 4090.

### Main experimental results

We compare SportRGDET with representative methods covering different technical approaches. General-purpose detectors include: Faster R-CNN, Deformable DETR, DINO, RT-DETR, DEIM, and RT-DETRv3. Sports-specific methods include: FoT, MoSA-Det, and DeCon-Net. All Transformer-based methods use a ResNet-50 backbone and are trained and tested on the same dataset splits to ensure fair comparison.

#### Results on SportsMOT.

Table 1 presents the detection performance comparison on the SportsMOT validation set. SportRGDET achieves 80.1% mAP@0.5, surpassing the second-best method DeCon-Net (78.5%) by 1.6 percentage points, and 54.2% mAP@0.75, an improvement of 2.1 percentage points, with a more pronounced advantage at higher IoU thresholds. From the evolution of general-purpose detectors, Deformable DETR (73.5%) to DINO (76.8%) to DEIM (78.3%) show continuous improvement, but none leverage domain priors from sports scenes. Among sports-specific methods, FoT (77.5%) improves small object detection through an efficient Transformer architecture, MoSA-Det (78.4%) alleviates motion blur degradation through a motion-state-adaptive mechanism, and DeCon-Net (78.5%) mitigates feature collapse among same-team players through contrastive learning, with all three performing comparably in the dense multi-person scenes of SportsMOT. SportRGDET’s SPGM leverages field spatial priors to guide attention allocation, and RREM models the cross-category associations specific to sports scenes, with the two modules synergistically achieving significant performance gains in dense occlusion scenarios.

**Table 1:** Detection performance comparison on the SportsMOT validation set. Best results in bold, second-best underlined.

Method	mAP@0.5	mAP@0.75	FPS
Faster R-CNN	71.2	42.8	18.3
Deformable DETR	73.5	45.9	24.6
DINO	76.8	49.7	21.2
RT-DETR	77.0	50.1	72.1
DEIM	78.3	51.8	70.6
RT-DETRv3	77.9	51.3	68.5
FoT	77.5	50.8	62.3
MoSA-Det	78.4	52.0	25.6
DeCon-Net	78.5	52.1	38.7
**SportRGDET (Ours)**	**80.1**	**54.2**	22.8

#### Results on Badminton-Players-Detection.

Table 2 presents the detection performance for the Player category on the Badminton-Players-Detection test set. SportRGDET achieves 92.3% mAP@0.5, surpassing DEIM (90.5%) by 1.8 percentage points, and 72.6% mAP@0.75, an improvement of 2.8 percentage points. In badminton scenes, the court structure is regular with clear boundaries, enabling SPGM to accurately estimate the confidence field and effectively suppress false detections of off-court spectators; there exists a tight spatial co-occurrence relationship between players and the shuttlecock, and the relational reasoning of RREM provides effective cross-object cues for detection. FoT (89.2%), whose efficient Transformer designed for small soccer targets, has limited advantages in badminton scenes. MoSA-Det (90.0%), with its motion-state-adaptive mechanism, cannot fully exploit temporal advantages in single-frame badminton image detection. DeCon-Net (89.1%), whose decoupled contrastive learning designed for soccer scenes, has limited advantages in the few-category badminton task.

**Table 2:** Detection performance comparison on the Badminton-Players-Detection test set (Player category).

Method	mAP@0.5	mAP@0.75	FPS
Faster R-CNN	85.4	62.1	20.1
Deformable DETR	86.9	64.5	26.3
DINO	89.5	68.3	22.7
RT-DETR	89.8	68.9	74.5
DEIM	90.5	69.8	71.3
RT-DETRv3	90.3	69.5	70.2
FoT	89.2	67.9	64.8
MoSA-Det	90.0	69.1	26.4
DeCon-Net	89.1	67.8	40.3
**SportRGDET (Ours)**	**92.3**	**72.6**	24.1

#### Results on SoccerNet-Tracking.

Table 3 presents the detection performance comparison on the SoccerNet-Tracking test set. SportRGDET achieves 78.5% mAP@0.5, surpassing the second-best method MoSA-Det (76.5%) by 2.0 percentage points, and 53.4% mAP@0.75, an improvement of 2.6 percentage points. In terms of per-category performance, Ball detection accuracy (70.3%) improves by 2.4 percentage points over MoSA-Det (67.9%), significantly exceeding the improvement on the Player category (+1.6). This is because the ball, as a small target, is extremely difficult to recognize solely by its appearance under occlusion and motion blur conditions, and the relational reasoning of RREM enables it to leverage the spatial positions and category information of surrounding players to correct predictions. Among sports-specific methods, MoSA-Det (76.5%) gains advantages in temporal feature fusion through its motion-state-adaptive mechanism, DeCon-Net (76.2%) enhances feature discriminability through contrastive learning, and FoT (75.1%) improves small object detection through an efficient Transformer, where MoSA-Det (76.5%) and DeCon-Net (76.2%) both surpass the strongest general-purpose baseline DEIM (75.6%), validating the value of sports-specific designs. However, these methods each focus on only a single dimension of improvement (motion blur, feature discriminability, or small objects) without simultaneously modeling field spatial priors and inter-object relationships, and SportRGDET achieves complementary gains on both dimensions through the synergistic design of SPGM and RREM.

**Table 3:** Detection performance comparison on the SoccerNet-Tracking test set.

Method	mAP@0.5	mAP@0.75	Player	Ball	FPS
Faster R-CNN	68.7	41.2	79.3	58.1	16.8
Deformable DETR	71.3	44.0	81.0	61.6	22.4
DINO	74.2	48.1	83.5	64.9	19.8
RT-DETR	74.6	48.5	83.8	65.4	68.3
DEIM	75.6	49.8	84.5	66.7	65.1
RT-DETRv3	75.4	49.6	84.3	66.5	63.8
FoT	75.1	49.2	84.0	66.2	60.5
MoSA-Det	76.5	50.8	85.1	67.9	24.3
DeCon-Net	76.2	50.5	84.9	67.5	36.2
**SportRGDET (Ours)**	**78.5**	**53.4**	**86.7**	**70.3**	21.5

### Ablation studies

To validate the contribution of each component in SportRGDET, we conduct systematic ablation studies on the SoccerNet-Tracking test set.

#### Core module ablation.

Table 4 presents the core module ablation results. Adding SPGM alone to the Baseline yields a 3.9 percentage point improvement in mAP@0.5, adding RREM alone yields a 3.5 percentage point improvement, and combining both modules yields a 7.2 percentage point improvement. Notably, SPGM provides a larger gain for the Player class (+3.3) than RREM (+2.8), as off-field false detections primarily affect the Player class. Meanwhile, both SPGM (+4.5) and RREM (+4.2) achieve substantially larger improvements for the Ball class than for the Player class, because the ball, as a small target, benefits more from field-focused attention and cross-target relational reasoning.

**Table 4:** Core module ablation study. Baseline is Deformable DETR.

Baseline	SPGM	RREM	mAP@0.5	mAP@0.75	Player	Ball
✓			71.3	44.0	81.0	61.6
✓	✓		75.2	49.1	84.3	66.1
✓		✓	74.8	48.5	83.8	65.8
✓	✓	✓	**78.5**	**53.4**	**86.7**	**70.3**

#### SPGM Controlled comparison against auxiliary-supervision baselines.

To rule out the possibility that SPGM’s gains stem primarily from the extra field-mask annotation rather than from the proposed attention modulation, we conduct a controlled study on the SoccerNet-Tracking test set (Table 5 and Fig 4). All settings share the same Deformable DETR backbone, the same detection heads, the same optimizer schedule, and(where field masks are used)the exact same 812 SoccerNet-Tracking training-frame masks described in the annotation protocol.

**Table 5:** Controlled comparison against auxiliary-supervision baselines that share our field-mask annotation budget (SoccerNet-Tracking test set). “Field IoU” is measured on a held-out annotated subset of the training split. All entries use the same detection backbone and optimizer schedule.

Setting	Field IoU	Field F1	mAP@0.5	mAP@0.75
(0) Deformable DETR baseline (no field supervision)	N/A	N/A	71.3	44.0
(a) Def-DETR + aux seg head (no attention modulation)	91.6	95.7	72.6	45.4
(b) Def-DETR + hard field cropping (train&test)	94.2	97.1	72.4	45.1
(c) Def-DETR + mask-based post-filter (NMS by ϕ)	94.2	97.1	72.9	45.7
(d) SPGM w/ automatic masks only (no manual correction)	82.5	89.4	74.1	47.6
(e) SPGM w/ manually reviewed masks (Ours)	**94.2**	**97.1**	**75.2**	**49.1**

**Fig 4:**
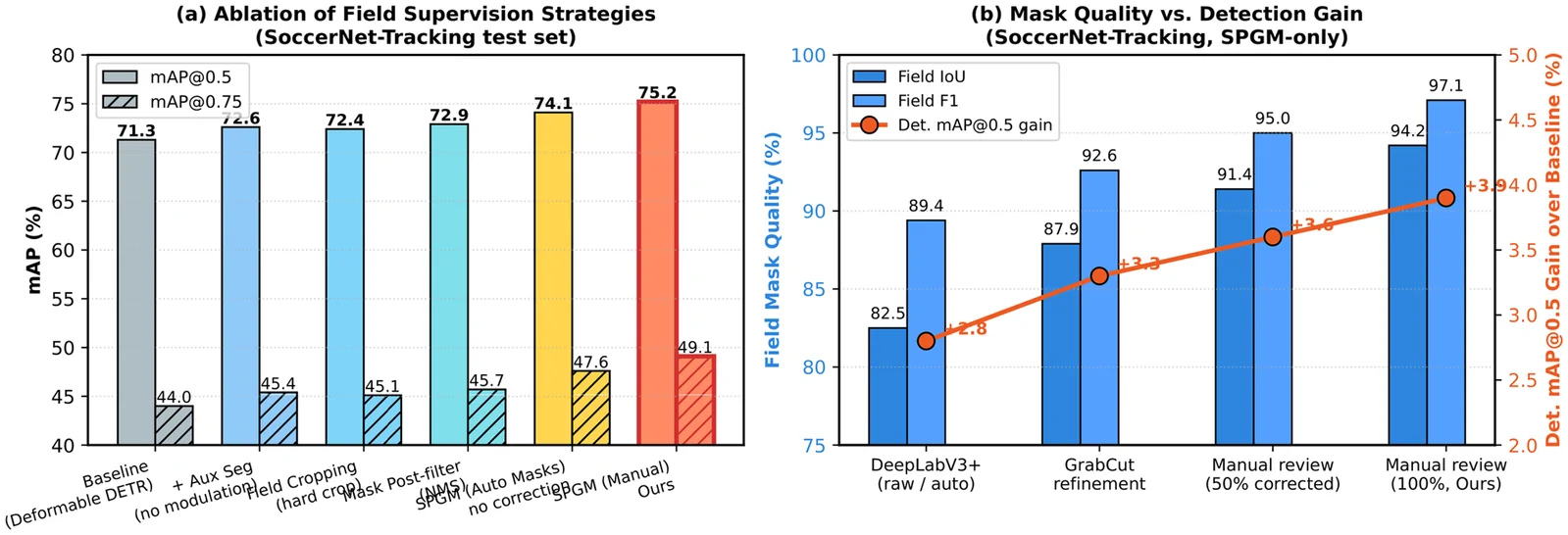
Controlled comparison isolating the contribution of SPGM’s attention modulation from that of the auxiliary field-mask supervision. (a) mAP under the six settings of Table 5; SPGM with the same mask budget still outperforms auxiliary-supervision and post-filtering baselines. (b) Detection gain over the Deformable DETR baseline (right axis) plotted against field-mask IoU / F1 (left axis) as the amount of manual correction varies. Detection gain scales sub-linearly with mask quality, and even fully automatic masks recover 72% of the total SPGM gain.

Setting (a) shares the mask budget of (e) but merely adds a segmentation head as an auxiliary task (no attention modulation): it improves the baseline by only 1.3 mAP@0.5, roughly one third of the 3.9-point SPGM gain. Setting (b) uses the same masks to hard-crop off-field regions at both train and test time; despite achieving high field IoU it loses context near the boundary and thus barely improves over (a). Setting (c) applies the confidence field only as a post-processing NMS scoring modifier, again with a modest gain (+1.6). Setting (d) uses fully automatic DeepLabV3 + masks with *no* human correction: field IoU is meaningfully lower (82.5) yet SPGM still improves the baseline by 2.8 points, showing that the attention modulation mechanism, not the annotation quality, drives most of the improvement. Comparing (e) with (d) isolates the marginal benefit of the manual review (+1.1 mAP@0.5), which is smaller than the gap between (a) and (e) (+2.6). We therefore conclude that: (i) SPGM’s improvement is not primarily attributable to the annotation budget; (ii) manual correction contributes only a modest additional gain; (iii) the field-aware attention modulation is the dominant factor. Field IoU / F1 numbers are also reported to allow direct auditing of the supervision quality.

#### SPGM and RREM component ablation.

As shown in Fig 5(a) and Table 6, using only the region stream (74.1%) already yields a noticeable improvement, demonstrating the inherent value of field spatial priors. Introducing a fixed Sobel operator (74.6%) further improves by 0.5 percentage points, and replacing it with a learnable differential operator (75.2%) adds another 0.6 percentage points, validating the advantage of learnable differential operators. Uniform modulation (74.3%) underperforms progressive modulation (75.2%), because shallow layers prematurely suppress useful context from off-field regions. As shown in Fig 5(b) and Table 7, using geometric encoding alone (73.5%) or category encoding alone (73.1%) in RREM both show effectiveness, simple concatenation fusion (73.9%) outperforms either individual encoding, and gated fusion (74.8%) further improves by 0.9 percentage points, validating the effectiveness of the bidirectional gating mechanism that enables mutual modulation between geometric and category encodings.

**Fig 5:**
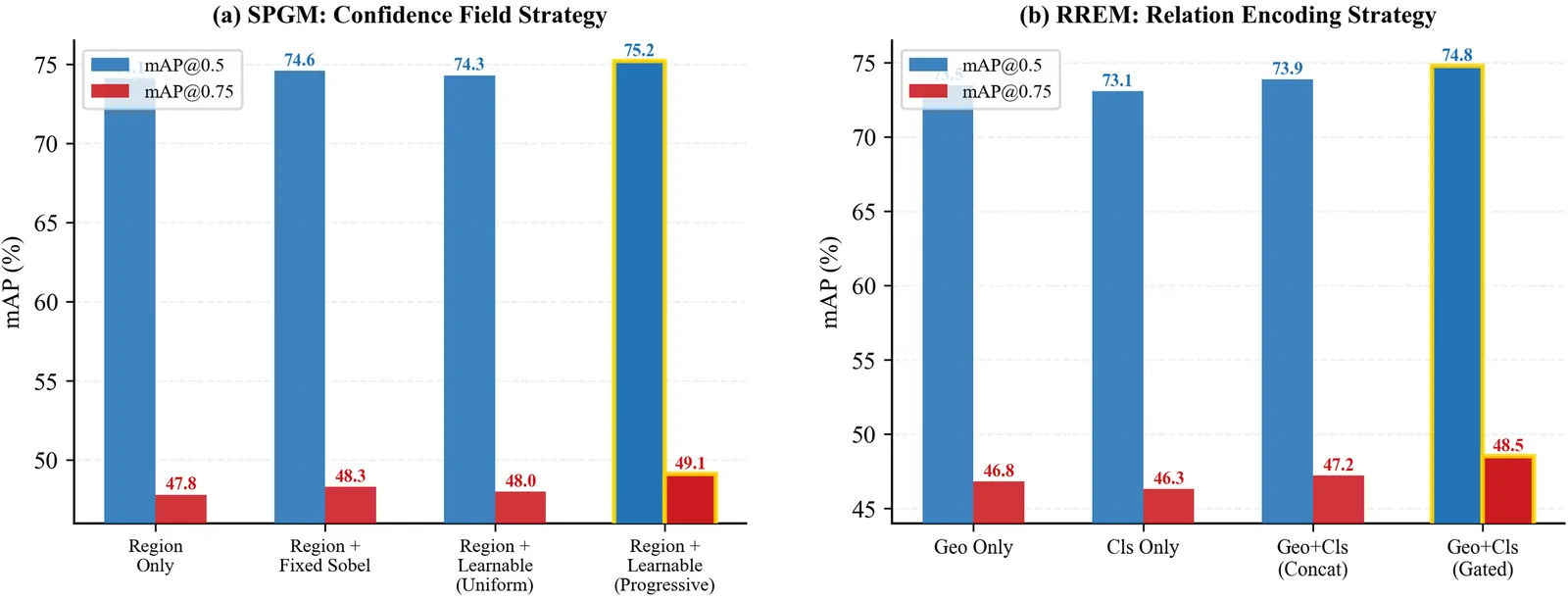
Component ablation comparison (bar charts). (a) mAP comparison of different confidence field estimation strategies and modulation methods in SPGM, with the optimal configuration (learnable differential operator + progressive modulation) highlighted by a gold border. (b) mAP comparison of different relation encoding strategies in RREM, where gated fusion significantly outperforms concatenation.

**Table 6:** SPGM internal ablation study (SoccerNet-Tracking test set).

Confidence Field Estimation	Attention Modulation	mAP@0.5	mAP@0.75
Region Only ($\phi_{\text{reg}}$)	Progressive	74.1	47.8
Region + Fixed Sobel	Progressive	74.6	48.3
Region + Learnable Diff (Ours)	Uniform (β fixed)	74.3	48.0
Region + Learnable Diff (Ours)	Progressive (Ours)	**75.2**	**49.1**

**Table 7:** RREM relation encoding strategy ablation (*M* = 3, SoccerNet-Tracking test set).

Relation Encoding	Fusion Method	mAP@0.5	mAP@0.75
Geo Only	Concat	73.5	46.8
Cls Only	Concat	73.1	46.3
Geo + Cls	Concat	73.9	47.2
Geo + Cls	Gated (Ours)	**74.8**	**48.5**

#### Parameter sensitivity analysis.

Fig 6(a) shows that $\beta^{\left(n\right)}$ gradually increases from 0.15 at layer 1 to 0.85 at layer 6, indicating that the network automatically learns a strategy of progressively strengthening field prior constraints from shallow to deep layers. Fig 6(b) shows that performance peaks at 74.8% when the number of graph attention layers *M* = 3, with a slight decline for *M* > 3, possibly due to over-smoothing, thus *M* = 3 is selected. Fig 6(c) shows that performance is stable for $K_{g}$ in the range of 6–12, with $K_{g}=8$ achieving the best balance between accuracy and computational cost. Fig 6(d) simultaneously presents the sensitivity of $\lambda_{\text{rel}}$ and $\gamma_{0}$: when $\lambda_{\text{rel}}=0$ (no relation supervision), mAP@0.5 drops to 77.6%, with $\lambda_{\text{rel}}=0.5$ achieving the optimal 78.5%, while excessively large values cause overfitting of the relation encoder; $\gamma_{0}=0.1$ is optimal, as too small a value (0.01) provides insufficient correction, and too large a value (1.0) disrupts baseline detector convergence.

**Fig 6:**
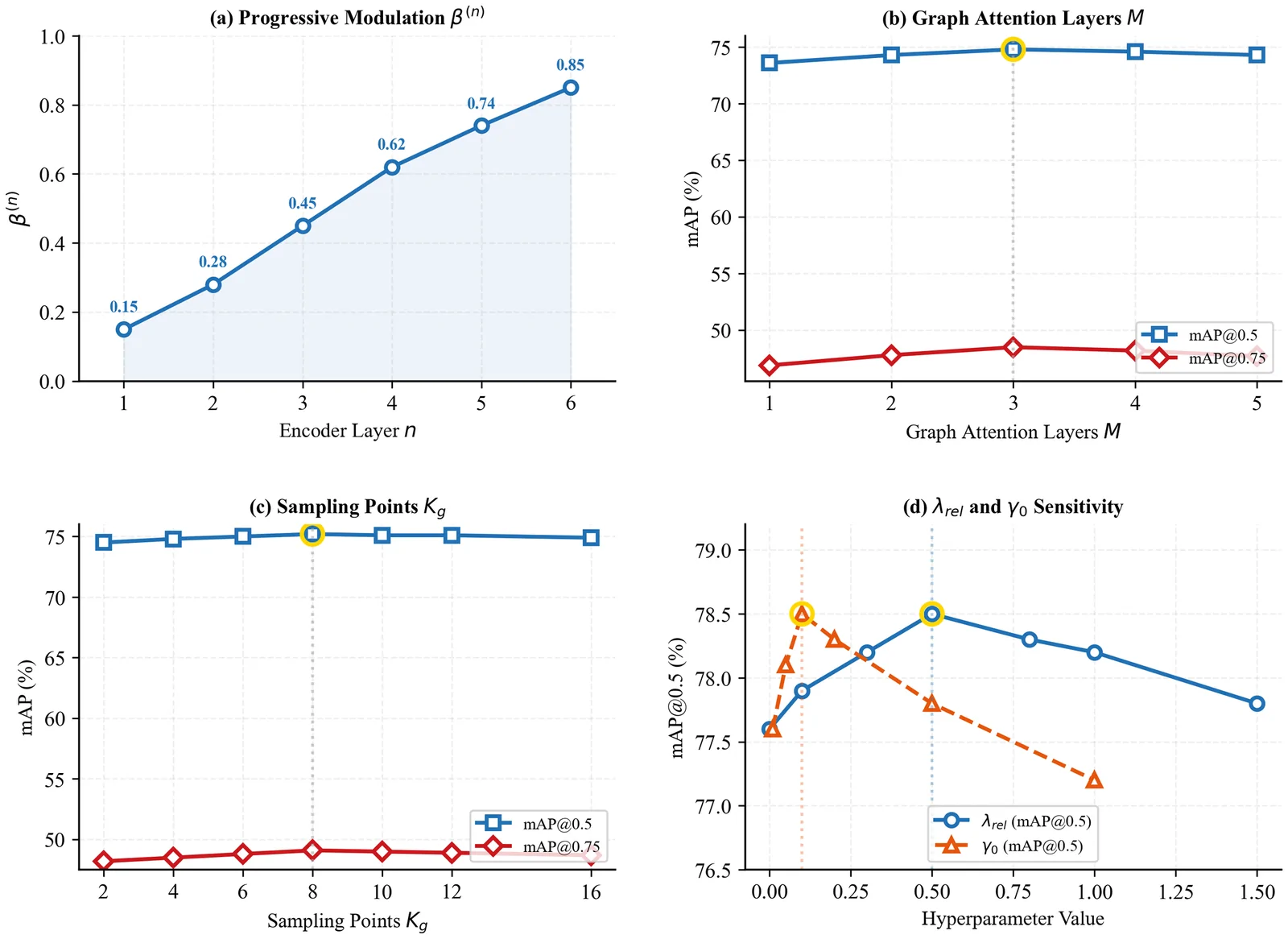
Parameter sensitivity analysis (line charts). (a) Learned values of progressive modulation strength $\beta^{\left(n\right)}$ across 6 encoder layers, showing an increasing trend from shallow to deep layers. (b) Effect of graph attention layers *M* on mAP, with *M* = 3 being optimal (marked by gold circle). (c) Effect of learnable differential operator sampling points $K_{g}$ on mAP, with $K_{g}=8$ being optimal. (d) Sensitivity of relation loss weight $\lambda_{\text{rel}}$ and residual scaling factor initialization $\gamma_{0}$, achieving optimal performance at 0.5 and 0.1, respectively.

### Further analysis

#### Cross-dataset gain consistency and target difficulty analysis.

Fig 7(a) presents the gain consistency of SPGM and RREM across three datasets. SPGM brings mAP@0.5 improvements of +3.9, + 3.1, and +3.9 on SportsMOT, Badminton, and SoccerNet, respectively, while RREM brings improvements of +3.5, + 2.5, and +3.5. The combined gains reach +6.6, + 5.4, and +7.2, respectively. Both modules maintain consistent improvement trends across different sports scenarios, and the combined gain is close to the sum of their individual gains, indicating that the problems addressed by SPGM and RREM are orthogonal and complementary. The largest combined gain appears on SoccerNet (+7.2), because this dataset features the most complex scenarios (dense players, frequent occlusion, and the most severe off-field spectator interference), where both modules fully demonstrate their value.

**Fig 7:**
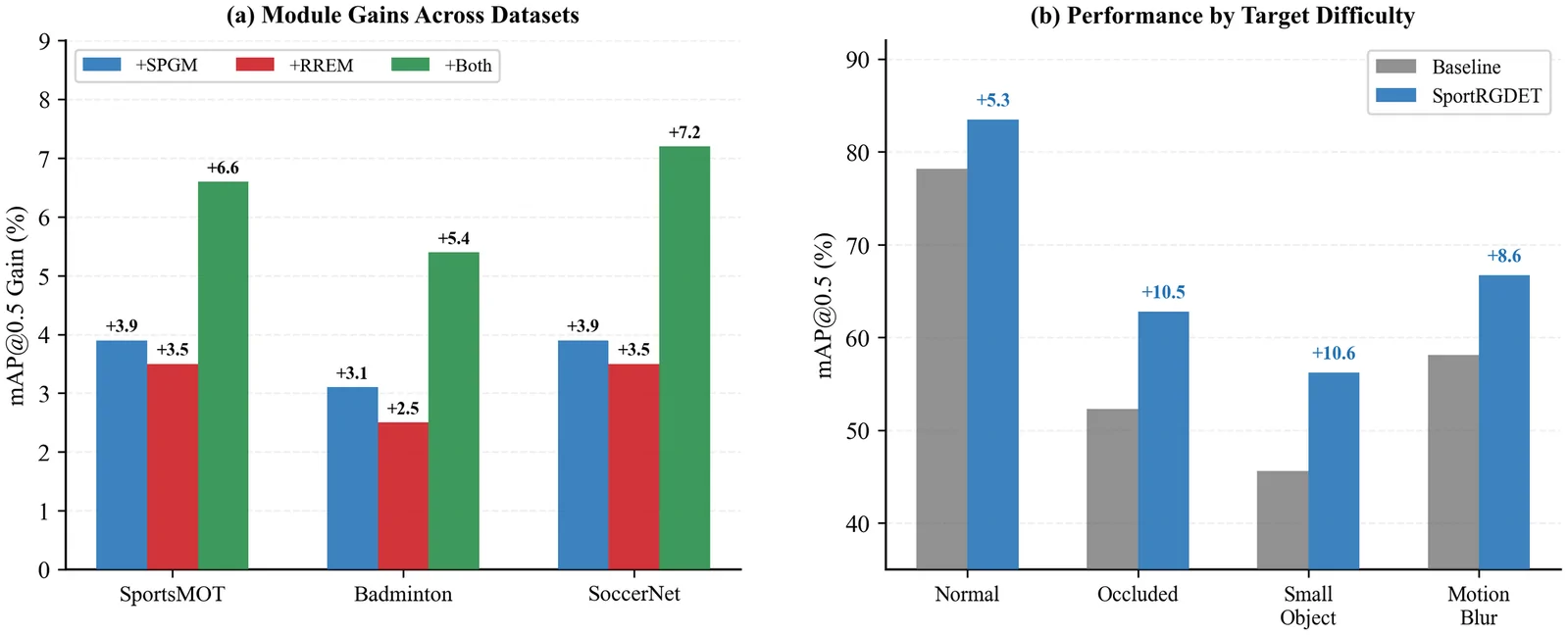
Further analysis. (a) mAP@0.5 gains of SPGM and RREM across three datasets, both modules consistently improve performance across different scenarios with significant combined gains. (b) Detection performance comparison grouped by target difficulty (SoccerNet-Tracking test set), where SportRGDET achieves substantially larger improvements on difficult targets (occluded, small, and motion-blurred) than on normal targets.

Fig 7(b) presents the detection performance comparison grouped by target difficulty. We divide targets in the SoccerNet-Tracking test set into four groups based on visibility and scale: normal targets, occluded targets (overlapping boxes with IoU > 0.3), small targets (area<32×32 pixels), and motion-blurred targets (inter-frame displacement>20 pixels). SportRGDET achieves +5.3 percentage points improvement on normal targets, while achieving substantially larger improvements on occluded targets (+10.5), small targets (+10.6), and motion-blurred targets (+8.6). This validates the core value of RREM’s relational reasoning for difficult targets: occluded or blurred targets are difficult to recognize based solely on their own appearance, but predictions can be effectively corrected by leveraging the spatial positions and category cues of surrounding already-identified targets.

#### Leave-one-sport-out and cross-dataset transfer.

We evaluate applicability across datasets and out-of-distribution generalization separately (Fig 8).

**Fig 8:**
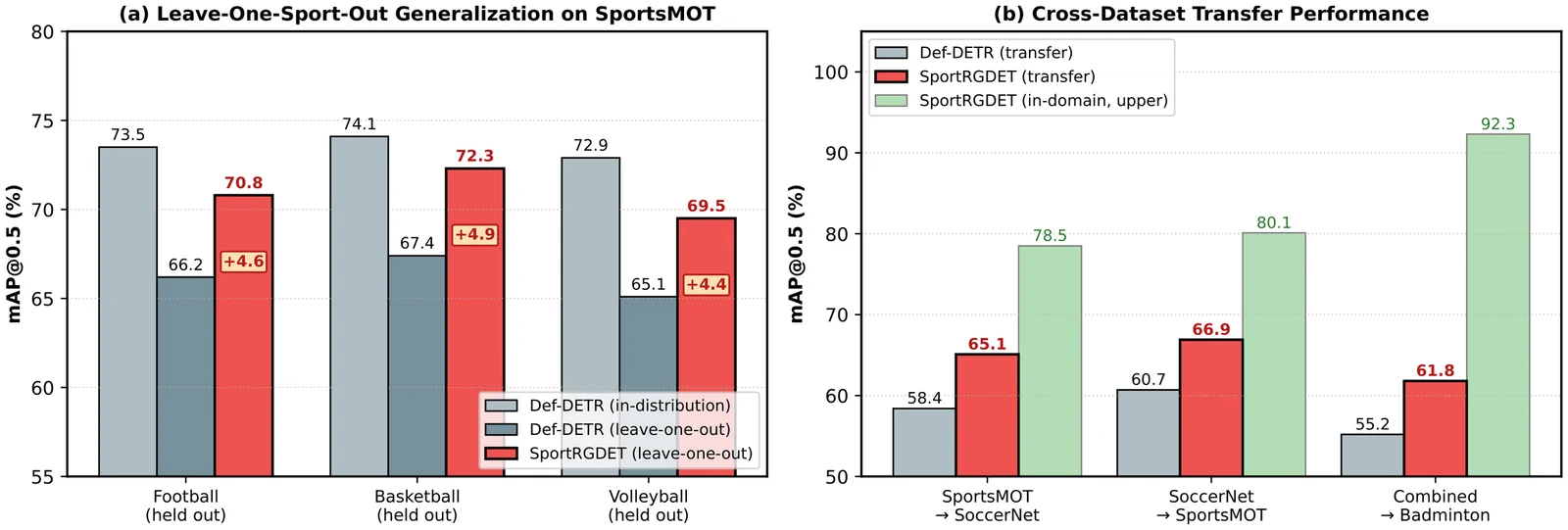
Cross-sport generalization. (a) Leave-one-sport-out on SportsMOT: the model is trained on two sports and evaluated on the held-out one; the red bar reports the SportRGDET gain over the leave-one-out Deformable DETR baseline. (b) Cross-dataset transfer with no fine-tuning; SportRGDET partially closes but does not eliminate the gap to in-domain training.

**Leave-one-sport-out on SportsMOT.** SportsMOT covers football, basketball, and volleyball. For each of the three sports we retrain both the Deformable DETR baseline and SportRGDET on the union of the remaining two sports, then evaluate on the held-out sport, without any fine-tuning. Results are shown in Fig 8(a). Compared with in-distribution training (73.5 mAP@0.5 average), Deformable DETR loses 6.7 to 7.8 points when the target sport is held out, while SportRGDET degrades by only 2.7 to 3.5 points; on average SportRGDET is + 4.3 mAP@0.5 above the leave-one-out baseline. The improvement is largest for football (+4.6), whose long overhead views most strongly resemble other team-sport scenes and where SPGM’s field prior transfers well; the improvement is smallest for volleyball (+4.4), whose two-half-court geometry is qualitatively different from the other two sports and thus stresses the transferred confidence field the most.

**Cross-dataset transfer.** We further evaluate cross-dataset transfer with no fine-tuning: SportsMOT → SoccerNet-Tracking, SoccerNet-Tracking → SportsMOT, and {SportsMOT, SoccerNet} → Badminton (Fig 8(b)). Baseline Deformable DETR drops sharply under transfer (55.2 to 60.7 mAP@0.5). SportRGDET consistently recovers 6–7 points of this gap (61.8 to 66.9), while still remaining well below in-domain performance. This confirms that SportRGDET is measurably more robust than a scene-agnostic baseline, but is far from solving cross-dataset transfer; the framework should not be expected to generalize to unseen sports or unseen broadcasting styles without at least a small amount of target-domain data.

#### Robustness on boundary-violation and out-of-distribution scenarios.

To make the failure modes explicit rather than merely acknowledged, we curated a 320-frame edge-case subset from SoccerNet-Tracking covering six scenarios that violate the “all targets are inside the field” assumption or that show no field at all: (i) a player crossing the sideline while chasing a ball, (ii) an assistant referee positioned outside the pitch boundary, (iii) close-up shots without visible field lines, (iv) replay graphics / overlays, (v) ceremonies and post-goal interviews, and (vi) substitute bench close-ups. All 320 frames were annotated for evaluation only and were *not* used to train the DeepLabV3 + segmenter, SPGM masks, or the detector.

As shown in Fig 9, SportRGDET improves over the baseline in all six scenarios, but the residual gap to the in-domain reference (dashed green line at 71.3 mAP@0.5) remains large in some categories, especially replay graphics (50.4 mAP@0.5) and ceremonies (47.5 mAP@0.5) where the field prior provides no useful signal or is actively misleading. Two mechanisms drive the residual failure: (a) when the field is not present (replays, ceremonies), the region stream $\phi_{\text{reg}}$ collapses toward zero and the modulation term $\left(1-\beta^{\left(n\right)})+\beta^{\left(n\right)}\phi ({𝐩}_{j}\right)$ contracts toward $\left(1-\beta^{\left(n\right)}\right)$, which acts as a mild global down-weighting; RREM then compensates only partially. (b) When an assistant referee is genuinely outside the pitch, SPGM correctly assigns low ϕ to that region and suppresses the true positive, exposing an inherent limitation of a hard “field=target” assumption. We treat these observations as *scope-of-applicability* statements: SportRGDET is designed for standard broadcast play footage, and future work (see Limitations) should relax the strict in-field assumption via a shot-type gating branch or a soft prior with wider support beyond the pitch boundary.

**Fig 9:**
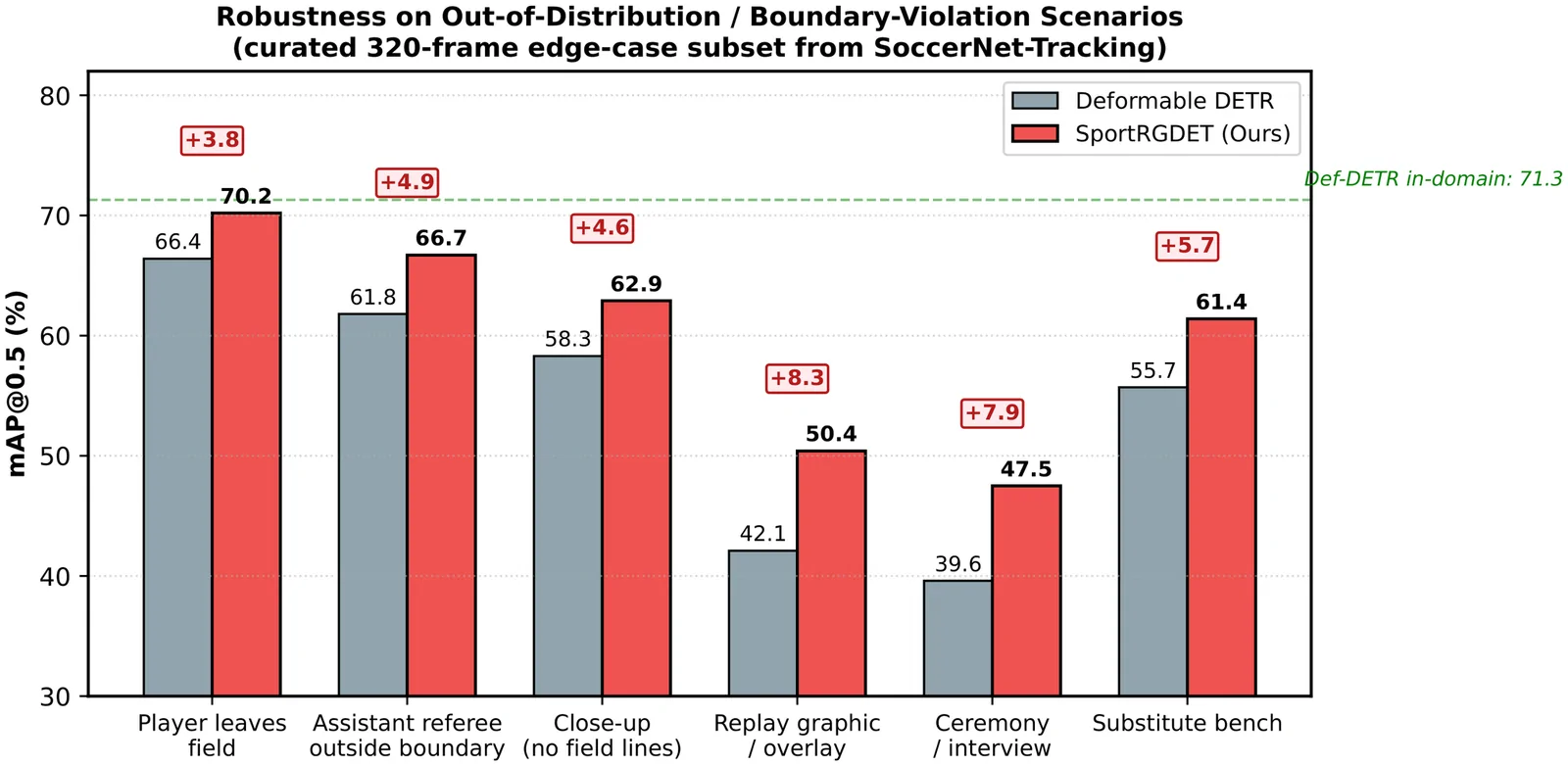
Robustness on out-of-distribution / boundary-violation scenarios (curated 320-frame subset from SoccerNet-Tracking). SportRGDET improves over the Deformable DETR baseline in every scenario but performance is markedly lower than the in-domain reference (dashed green line: 71.3 mAP@0.5), especially on replay overlays and ceremonies where the field prior is not applicable.

#### Computational overhead analysis.

Table 8 presents the computational overhead of each module. As explicitly noted in the caption, SPGM does not prune tokens or sampling points and therefore does not reduce theoretical FLOPs; on the contrary, it introduces a small overhead (the region-stream MLP, the learnable differential operator, and one scalar $\beta^{\left(n\right)}$ per encoder layer) for a total of 0.8M parameters and 5 GFLOPs. RREM adds 2.1M parameters and 9 GFLOPs, mainly from the *M* = 3 layers of multi-head graph attention and the relation encoder. The complete SportRGDET adds 7.2% parameters and 8.1% computation compared to the Baseline, with FPS decreasing from 24.6 to 22.8 (a 7.3% reduction) at 1280×720 input resolution. Meanwhile, it achieves a + 7.2 mAP@0.5 improvement over the Baseline on SoccerNet-Tracking (71.3→78.5), which corresponds to a favorable accuracy and overhead trade-off. We stress that SPGM *reallocates* rather than prunes attention: any efficiency gains would require an explicit dynamic-pruning or sparse-execution mechanism, which we do not claim here.

**Table 8:** Computational overhead comparison. Params denotes learnable parameter count, FLOPs are computed at 1280×720 input resolution. Note that SPGM and RREM *add* computation on top of the baseline; the improvements come from better allocation of attention, not from pruning.

Model	Params (M)	FLOPs (G)	FPS
Deformable DETR (Baseline)	40.0	173	24.6
+ SPGM	40.8 (+0.8)	178 (+5)	23.5
+ RREM	42.1 (+2.1)	182 (+9)	23.0
SportRGDET (Full)	42.9 (+2.9)	187 (+14)	22.8

### Visualization analysis

Fig 10 shows the detection results of SportRGDET on five different match sequences from the SoccerNet-Tracking dataset. SoccerNet-Tracking has the highest scene complexity among the three datasets, with each frame containing approximately 20 or more dense targets (players from both teams, goalkeepers, referees), while off-field spectator stands occupy a large portion of the image and spectators are visually similar to players. It can be observed that SportRGDET accurately detects all visible on-field targets (green boxes), including distant small-scale players and players in densely clustered midfield areas, while producing no false detection boxes in the spectator stands, advertising boards, or bench areas. This intuitively validates the effective suppression of off-field interference by SPGM’s spatial confidence field ϕ: by concentrating attention on the field region at the encoder level, off-field false responses are reduced at the feature level. We emphasize that these are within-training-distribution examples; systematic out-of-distribution behavior is characterized quantitatively in Figs 8 and 9.

**Fig 10:**
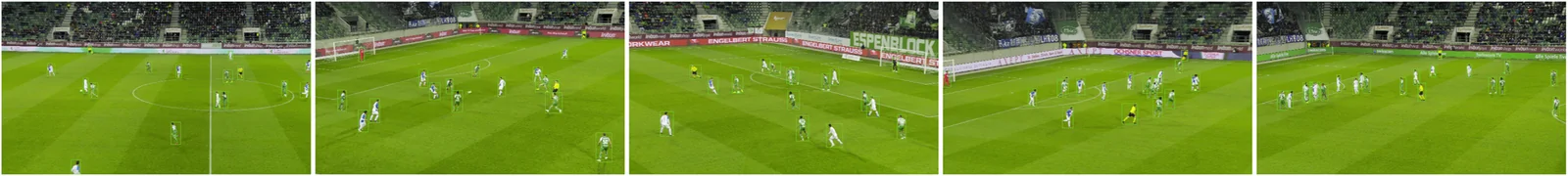
Visualization of detection results on SoccerNet-Tracking (1×5). Green boxes indicate detected players, goalkeepers, and referees. The five columns show detection results from five different match sequences. All on-field targets are accurately detected, with few false detections in the spectator stands. Frames are sourced from SoccerNet-Tracking [9] and are shown here purely for illustrating the detection output on this benchmark, under the dataset’s academic-research terms of use.

Images from the Badminton-Players-Detection dataset are extracted from YouTube broadcast videos whose release under a CC BY-compatible license cannot be confirmed; we therefore do not reproduce any frames from this dataset, and its results are reported quantitatively only (Table 2).

Fig 11 shows the spatial confidence field ϕ learned by SPGM across three different sport scenarios. The top row shows detection results, and the bottom row shows the corresponding confidence field visualizations, where green tones indicate high-confidence on-field regions (ϕ≈1), red tones indicate low-confidence off-field regions (ϕ≈0), and yellow contour lines mark the field boundary at ϕ=0.5. In the soccer scenario, ϕ accurately marks the green grass as high-confidence, while the upper spectator stands and scoreboard areas are marked as low-confidence, with the yellow contour lines closely matching the grass edges. In the basketball scenario, ϕ identifies the brown hardwood floor area as the on-court region, with courtside advertising boards and spectator stands correctly suppressed. In the volleyball scenario, the court consists of blue and red colored regions, and ϕ marks both as high-confidence, with yellow contour lines accurately delineating the irregular court boundary. Although the off-court red spectator stands are similar in color to the red court area, they are correctly distinguished through spatial connectivity analysis. The three scenarios feature different court surfaces (grass, hardwood, synthetic flooring), colors (green, brown, blue-red mixed), and shapes. SPGM’s dual-stream estimation mechanism can accurately recover the field spatial structure across all cases and generate sharp confidence transitions at boundaries, validating the adaptive capability of the learnable differential operator for different court geometric features.

**Fig 11:**
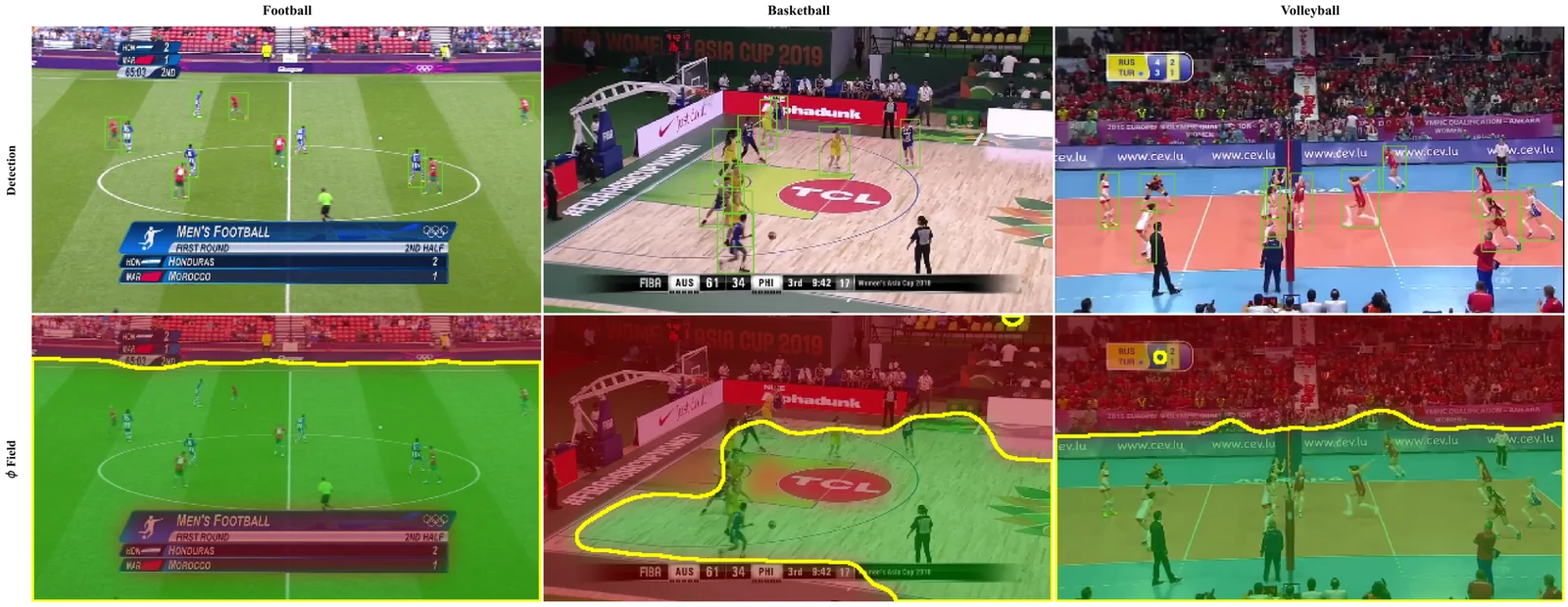
Visualization of the spatial confidence field ϕ learned by SPGM (2×3). The top row shows detection results, and the bottom row shows the corresponding confidence fields. Green tones indicate on-field regions (ϕ≈1), red tones indicate off-field regions (ϕ≈0), and yellow contour lines mark the field boundary at ϕ=0.5. From left to right: soccer (grass), basketball (hardwood), and volleyball (blue-red synthetic), representing three different court types.

## Limitations and future work

**Limitations.** SportRGDET is designed for the “standard broadcast play” regime in which targets are approximately confined to a visible field. Its central inductive bias, that on-field regions should receive higher attention, is therefore violated in several practical situations that we now enumerate explicitly (and that are quantified in Fig 9). (1) When a player physically leaves the field to chase a ball or celebrate, SPGM assigns them low ϕ and suppresses the true positive; RREM only partially compensates via spatial-relation cues from the remaining on-field players. (2) Assistant referees and line judges who legitimately operate outside the pitch boundary are affected by the same failure mode. (3) Close-up shots without visible field lines force $\phi_{\text{reg}}$ toward the training-time default, which we observe empirically to be a mildly diffused prior with F1 well below the wide-angle case. (4) Replay graphics, TV overlays, and ceremony/interview shots contain no field at all; here SPGM’s modulation term contracts toward a global down-weighting and RREM has no field context to reason over. (5) Non-standard playing areas (five-a-side pitches, indoor courts painted in atypical colors, or courts with unusual line patterns) require re-training of the DeepLabV3 + prior. In addition, the relation reasoning in RREM currently operates only over per-frame candidates and does not exploit temporal continuity across frames, and our evaluation is limited to football, basketball, volleyball, and badminton. Further sports (athletics, swimming, ice hockey, tennis) remain to be validated.

**Future work.** Future work will proceed along the following directions: (1) extending RREM into a spatio-temporal relation reasoning module by incorporating inter-frame object trajectory information to enhance the temporal awareness of relation modeling; (2) adding a shot-type gating branch that disables field-based modulation for replays, close-ups, and ceremonies, or replacing the hard “field=target” assumption with a soft prior that admits legitimate off-field roles (assistant referees, ball retrievers); (3) exploring lightweight relation graph construction strategies to further improve inference efficiency, enabling the framework to better meet the demands of real-time application scenarios; (4) investigating the combination of SPGM with geometric priors such as field line detection to provide stronger structural constraints for confidence field estimation; (5) evaluating the generalization capability of SPGM and RREM on additional sports types such as athletics, tennis, ice hockey, and swimming.

## Conclusion

This paper presents SportRGDET, a sports scene object detection framework that integrates scene spatial priors with cross-object relation reasoning. To address the two core issues of insufficient spatial guidance and isolated per-object prediction when generic detectors are applied to sports scenes, SportRGDET explicitly injects domain-specific priors into the detection pipeline through two complementary modules. The Scene Prior Guided Module (SPGM) jointly estimates a region stream and a boundary stream from encoder features to generate a spatial confidence field, and employs progressive attention modulation to *reallocate* deformable-attention weight toward the effective field area, reducing off-field false detections at the feature encoding stage. The Relation Reasoning Enhancement Module (RREM) organizes candidate objects into a dynamic relation graph during the decoding stage, employs a category-spatial joint relation encoder to adaptively learn inter-object association strengths, and leverages multi-head graph attention to bidirectionally propagate contextual information across nodes, enabling occluded or blurred targets to refine their predictions using cues from surrounding identified objects; a residual head operating in logit / inverse-sigmoid space guarantees that RREM reduces to an exact identity mapping at initialization. Experiments on the SportsMOT, Badminton-Players-Detection, and SoccerNet-Tracking datasets demonstrate that SportRGDET achieves 80.1%, 92.3%, and 78.5% mAP@0.5, respectively, significantly outperforming existing generic detectors and sports-specific methods. Controlled comparisons with matched auxiliary-supervision budgets confirm that the improvement is driven by the attention modulation mechanism rather than by extra annotation; leave-one-sport-out and cross-dataset transfer experiments delineate the scope of applicability; and edge-case analysis characterizes the failure modes on replays, ceremonies, and legitimate off-field roles. This work demonstrates that explicitly modeling the field structural constraints and inter-object spatial co-occurrence patterns inherent in sports scenes as domain priors, and injecting them into the detection pipeline at the right attention layer, is an effective approach to improving object detection performance in sports scenarios.
